# Supplementary material for: Brown and beige adipose tissue regulate systemic metabolism through a metabolite interorgan signaling axis
Source: Nat Commun. 2021 Mar 26;12:1905. doi: 10.1038/s41467-021-22272-3 (PMC7998027; doi:10.1038/s41467-021-22272-3)
Supplement: Supplementary file 1 — Supplementary Information [file 41467_2021_22272_MOESM1_ESM.pdf]

Supplementary Information

Brown and beige adipose tissue regulate systemic metabolism through a metabolite interorgan signaling axis

Anna Whitehead[1‡], Fynn N Krause[2‡], Amy Moran[1‡], Amanda D.V. MacCannell[1], Jason L Scragg[1], Ben D McNally[2], Edward Boateng[1], Steven A Murfitt[2], Samuel Virtue[3], John Wright[1], Jack Garnham[1], Graeme R Davies[4], James Dodgson[5], Jurgen E Schneider[1], Andrew J Murray[6], Christopher Church[4], Antonio Vidal-Puig[3], Klaus K Witte[1], Julian L Griffin[2], Lee D Roberts[1*]

1. School of Medicine, University of Leeds, Leeds LS2 9JT, UK.

2. Department of Biochemistry, University of Cambridge, Cambridge CB2 1GA, UK.

3. Institute of Metabolic Science, University of Cambridge, Cambridge CB2 0QQ, UK.

4. Bioscience Metabolism, Research and Early Development, Cardiovascular, Renal and Metabolism, BioPharmaceuticals R&D, AstraZeneca, Cambridge, UK

5. Phenotypic Screening and High Content Imaging, Antibody Discovery & Protein Engineering, R&D, AstraZeneca, Cambridge, UK

6. Department of Physiology, Development and Neuroscience, University of Cambridge, Cambridge CB2 3DY, U.K.

[‡] Equal Contribution

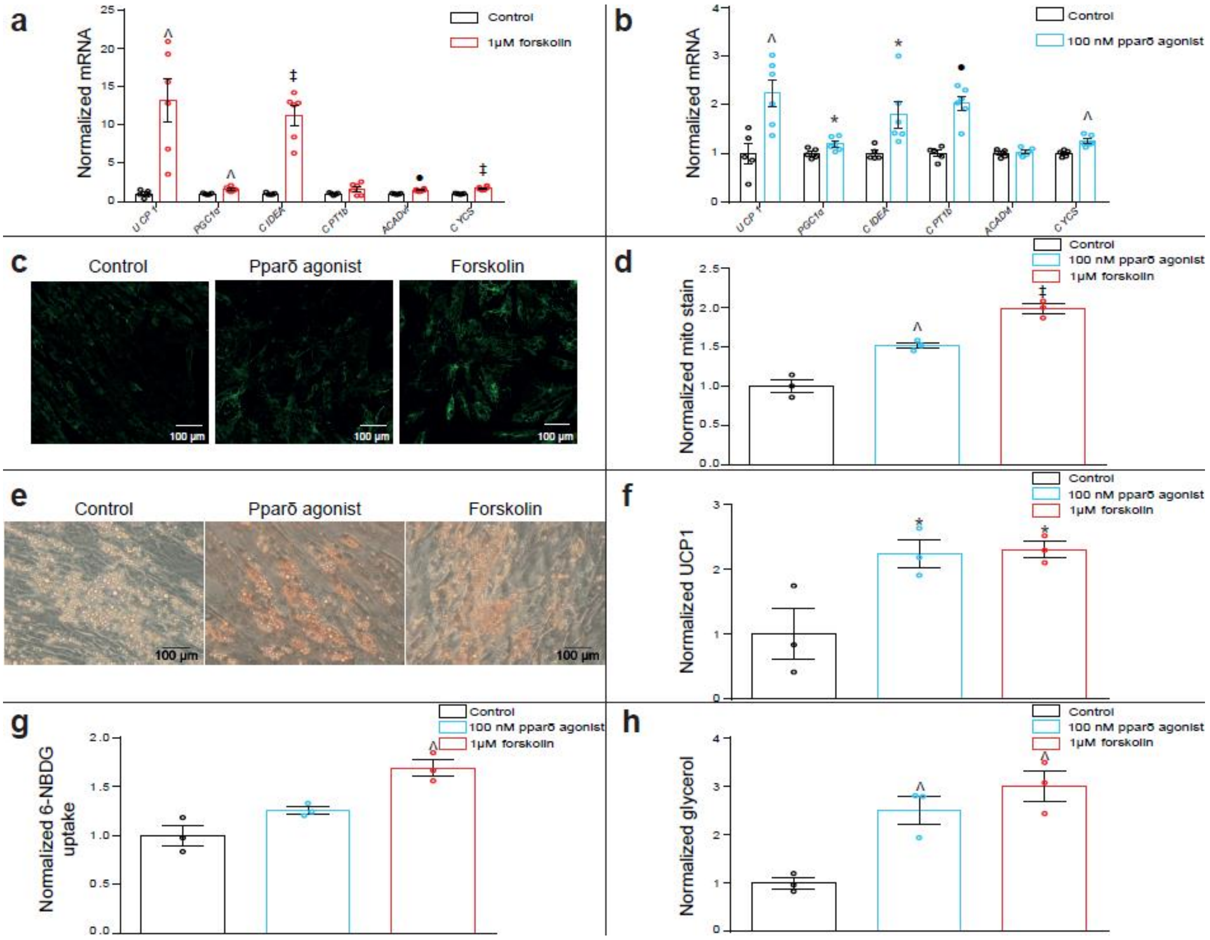

Supplementary Figure 1. Forskolin and PPARδ-agonism induce browning of human primary adipocytes

(**a**) Forskolin (1 μM) increases the expression of brown adipocyte-associated genes in primary human adipocytes (Control n = 5, Forskolin n = 6; two-tailed t-test; *UCP1 p* = 0.0035, *PGC1α p* = 0.0023, *CIDEA p* = 0.000048, *ACADvl p* = 0.00014, *CYCS p* = 0.000055). (**b**) PPARδ agonist GW0742 induces expression of brown adipocyte-associated genes in primary human adipocytes Control n = 5, PPARδ agonist n = 6; two-tailed t-test; *UCP1 p* = 0.007, *PGC1α p* = 0.026, *CIDEA p* = 0.027, *CPT1b p* = 0.00018, *CYCS p* = 0.0017). (**c**) Mitotracker Green staining of mitochondria in human primary adipocytes treated with a PPARδ agonist or forskolin (representative images of 3 independent repeats; scale bars = 100 μm). (**d**) Forskolin and PPARδ agonism increase mitochondrial content of human primary adipocytes (n = 3; One-way ANOVA with Dunnett's post hoc; PPARδ agonist *p* = 0.0021, Forskolin *p* < 0.0001). (**e**) Immunohistochemical staining of UCP1 (red) in human primary adipocytes treated with forskolin or PPARδ agonist GW0742 (representative images of 3 independent repeats; scale bars = 100 μm). (**f**) Forskolin and PPARδ agonism increase UCP1 protein content in human primary adipocytes (n = 3; One-way ANOVA with Dunnett's post hoc; PPARδ agonist *p* =

0.029, Forskolin *p* = 0.024). (**g**) Glucose uptake measured using fluorescent 6-NBDG in human primary adipocytes treated with forskolin or a PPARδ agoinst (n = 3; One-way ANOVA with Dunnett's post hoc; Forskolin *p* < 0.0015). (**h**) Extracellular glycerol as a measure of lipolysis analyzed using gas chromatography-mass spectrometry from the media of forskolin and PPARδ agonist treated human primary adipocytes (n = 3; One-way ANOVA with Dunnett's post hoc; PPARδ agonist *p* = 0.009, Forskolin *p* = 0.0024). Blue = PPARδ agonist, Red = Forskolin. ∗p ≤ 0.05, ^p ≤ 0.01, •p ≤ 0.001, ‡p ≤ 0.0001. Data are mean ± SEM with data points shown. Source data are provided as a Source Data file.

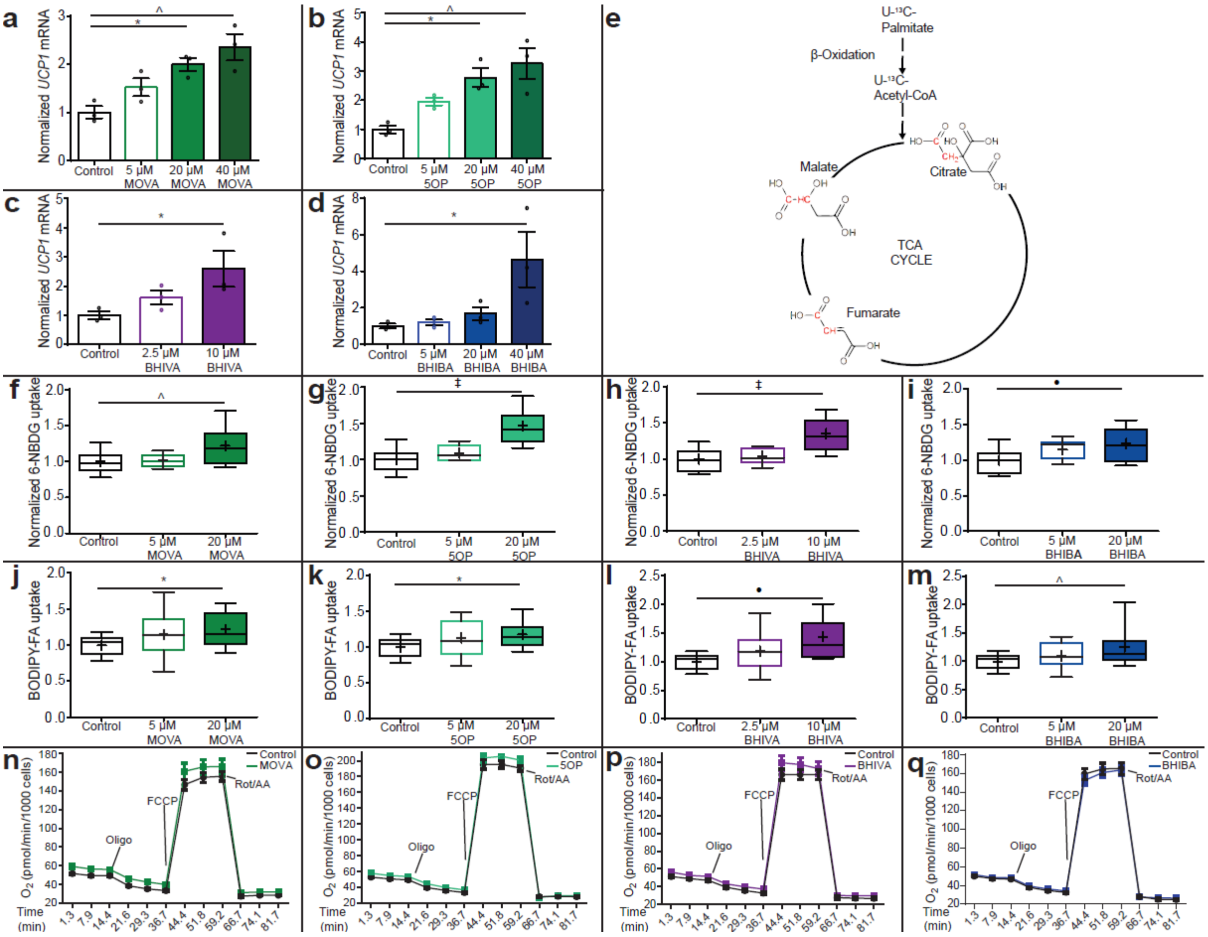

Supplementary Figure 2. Metabolites secreted from browning human primary adipocytes induce a brown adipocyte-like phenotype

UCP1 expression in primary human adipocytes treated with (**a**) 3-methyl-2-oxovaleric acid (MOVA; 10 µM, 20 µM, 40 µM; n = 3; One-way ANOVA with Dunnett's post hoc; 20 µM p = 0.015, 40 µM p = 0.0026), (**b**) 5-oxoproline (5OP; 10 µM, 20 µM, 40 µM; n = 3; One-way ANOVA with Dunnett's post hoc; 20 µM p = 0.012, 40 µM p = 0.003), (**c**) β-hydroxyisovaleric acid (BHIVA; 5 µM, 10 µM; n = 3; One-way ANOVA with Dunnett's post hoc; 10 µM p = 0.048) and (**d**) β-hydroxyisobutyric acid (BHIBA; 10 µM, 20 µM, 40 µM; n = 3; One-way ANOVA with Dunnett's post hoc; 40 µM p = 0.028). (**e**) Schematic showing TCA cycle ^{13}C-enrichment from ^{13}C-palmitate metabolism in metabokine-treated human adipocytes. Red carbons represent ^{13}C-labeling. Glucose uptake in (**f**) MOVA (Control n = 26, 5 µM n = 10, 20 µM n = 29; One-way ANOVA with Dunnett's post hoc; 20 µM p = 0.0018), (**g**) 5OP (Control n = 32, 5 µM n = 7, 20 µM n = 28; One-way ANOVA with Dunnett's post hoc; 20 µM p < 0.0001), (**h**) BHIVA (Control n = 35, 2.5 µM n = 9, 10 µM n = 30; One-way ANOVA with Dunnett's post hoc; 10 µM p < 0.0001) and (**i**) BHIBA (Control n = 24, 5 µM n = 9, 20 µM n = 29; One-way ANOVA with Dunnett's post hoc; 20 µM p = 0.0009) treated human primary adipocytes. Fatty acid uptake in (**j**) MOVA (Control n = 23, 5 µM n = 14, 20 µM n = 18; One-way ANOVA with Dunnett's post

hoc; 20 μM *p* = 0.012), (**k**) 5OP (Control n = 23, 5 μM n = 43, 20 μM n = 32; One-way ANOVA with Dunnett's post hoc; 20 μM *p* = 0.023), (**l**) BHIVA ((Control n = 23, 2.5 μM n = 10, 10 μM n = 18; One-way ANOVA with Dunnett's post hoc; 20 μM *p* = 0.0002) and (**m**) BHIBA (Control n = 23, 5 μM n = 34, 20 μM n = 18; One-way ANOVA with Dunnett's post hoc; 20 μM *p* = 0.003) treated human primary adipocytes. Mitostress respirometry traces of immortalized human white preadipocytes isolated from neck fat and differentiated to mature adipocytes in the presence of (**n**) MOVA (20 μM; Control n = 26, MOVA n = 29), (**o**) 5OP (20 μM; Control n = 29, MOVA n = 28), (**p**) BHIVA (10 μM; Control n = 28, MOVA n = 30) and (**q**) BHIBA (20 μM; n = 29). Points in the assay at which oligomycin (Oligo), carbonyl cyanide 4-(trifluoromethoxy)phenylhydrazone (FCCP) and rotenone and antimycin A (Rot/AA) were added are shown. Dark green = MOVA, light green = 5OP, purple = BHIVA, dark blue = BHIBA. ∗p ≤ 0.05, ^p ≤ 0.01, •p ≤ 0.001, ‡p ≤ 0.0001. Bar graphs show mean ± SEM with data points shown. Box and whisker plots show 25th to 75th percentile (box) min to max (whiskers), mean (+) and median (-). Source data are provided as a Source Data file.

.

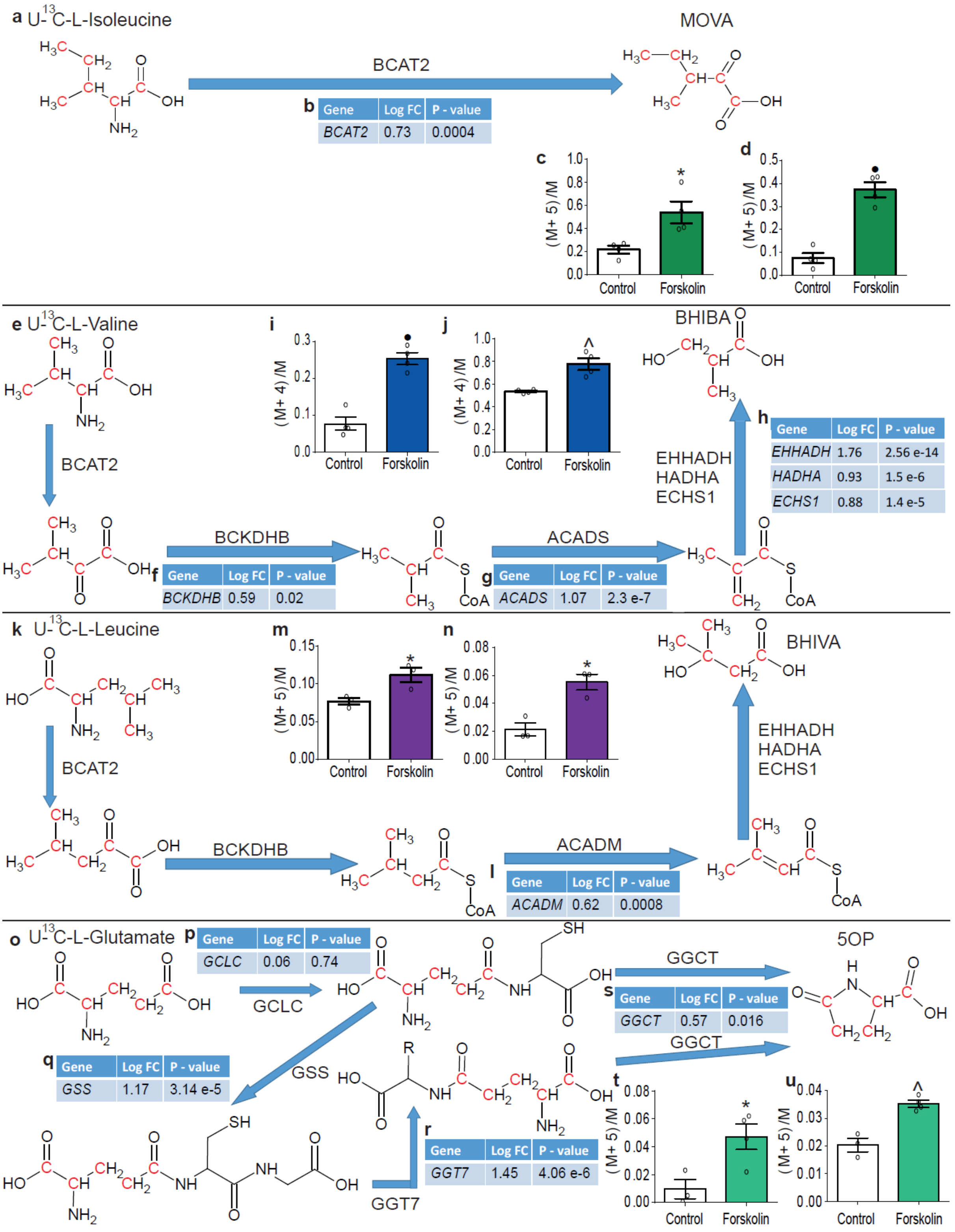

Supplementary Figure 3. ^{13}C-isotope substrate tracing and RNA-seq reveal mechanisms of MOVA, BHIBA, BHIVA and 5OP biosynthesis in browning human adipocytes

(**a**) Isoleucine metabolism to 3-methyl-2-oxovaleric acid (MOVA) showing ^{13}C-labeled substrates and products. (**b**) RNA-seq identifies increased expression of *branched chain*

amino acid transaminase 2 (*BCAT2*), which catalyses the reaction producing MOVA, in forskolin-treated primary human adipocytes. (**c**) Intracellular adipocyte ^{13}C-labeled MOVA (n = 4; *p* =0.018) and (**d**) extracellular ^{13}C-labeled MOVA (n = 4; *p* = 0.0003) in conditioned media from forskolin-treated human primary adipocytes incubated with ^{13}C-labeled isoleucine. (**e**) Valine metabolism to β-hydroxyisobutyric acid (BHIBA) showing ^{13}C-labeled substrates and products. RNA-seq of forskolin-treated adipocytes identifies increased expression of *BCAT2*, (**f**) *branched chain keto acid dehydrogenase E1 subunit beta* (*BCKDHB*), (**g**) *acyl-CoA dehydrogenase short chain* (*ACADS*), and (**h**) *Enoyl-Coenzyme A, Hydratase/3-Hydroxyacyl Coenzyme A Dehydrogenase* (*EHHADH*), *hydroxyacyl-CoA dehydrogenase (HADHA) and Enoyl-CoA Hydratase, Short Chain 1* (*ECHS1*), which catalyze the generation of BHIBA from valine. (**i**) Intracellular adipocyte ^{13}C-labeled BHIBA (n = 4; *p* = 0.0003) and (**j**) extracellular ^{13}C-labeled BHIBA (n = 4; *p* = 0.0039) in conditioned media from forskolin-treated human primary adipocytes incubated with ^{13}C-labeled valine. (**k**) Leucine metabolism to β-hydroxyisovaleric acid (BHIVA) showing ^{13}C-labeled substrates and products. RNA-seq of forskolin-treated adipocytes identifies increased expression of *BCAT2*, *BCKDHB*, (**l**) *acyl-CoA dehydrogenase medium chain* (*ACADM*), and *EHHADH*, *HADHA* and *ECHS1*, which catalyze the generation of BHIVA from valine. (**m**) Intracellular adipocyte ^{13}C-labeled BHIVA (n = 3; *p* = 0.0101) and (**n**) extracellular ^{13}C-labeled BHIVA (n = 3; *p* = 0.031) in conditioned media from forskolin-treated human primary adipocytes incubated with ^{13}C-labeled leucine. (**o**) Glutamate metabolism to 5-oxoproline (5OP) showing ^{13}C-labeled substrates and products. Expression of the 5OP biosynthetic enzymes (**p**) *glutamate-cysteine ligase catalytic subunit* (*GCLC*), (**q**) *glutathione synthetase* (*GSS*), (**r**) *γ-glutamyltransferase 7* (*GGT7*) and (**s**) *γ-glutamylcyclotransferase* (*GGCT*) from RNA-seq data of forskolin-treated human primary adipocytes. (**t**) Intracellular adipocyte ^{13}C-labeled 5OP (Control n = 3, 5OP n = 4; *p* = 0.028) and (**u**) extracellular ^{13}C-labeled 5OP (Control n = 3, 5OP n = 4; *p* = 0.002) in conditioned media from forskolin-treated human primary adipocytes incubated with 13C-labeled glutamate. ∗p ≤ 0.05, ^p ≤ 0.01, •p ≤ 0.001. Dark green = MOVA, light green = 5OP, purple = BHIVA, dark blue = BHIBA. ^{13}C in structures are labeled in red. Data in bar charts and tables was analysed with a two-tailed t-test. Bar charts are mean ± SEM with individual data shown. Data in tables are Log fold-change (Log FC) of three biological replicates p-values calculated by Exact test. Source data are provided as a Source Data file.

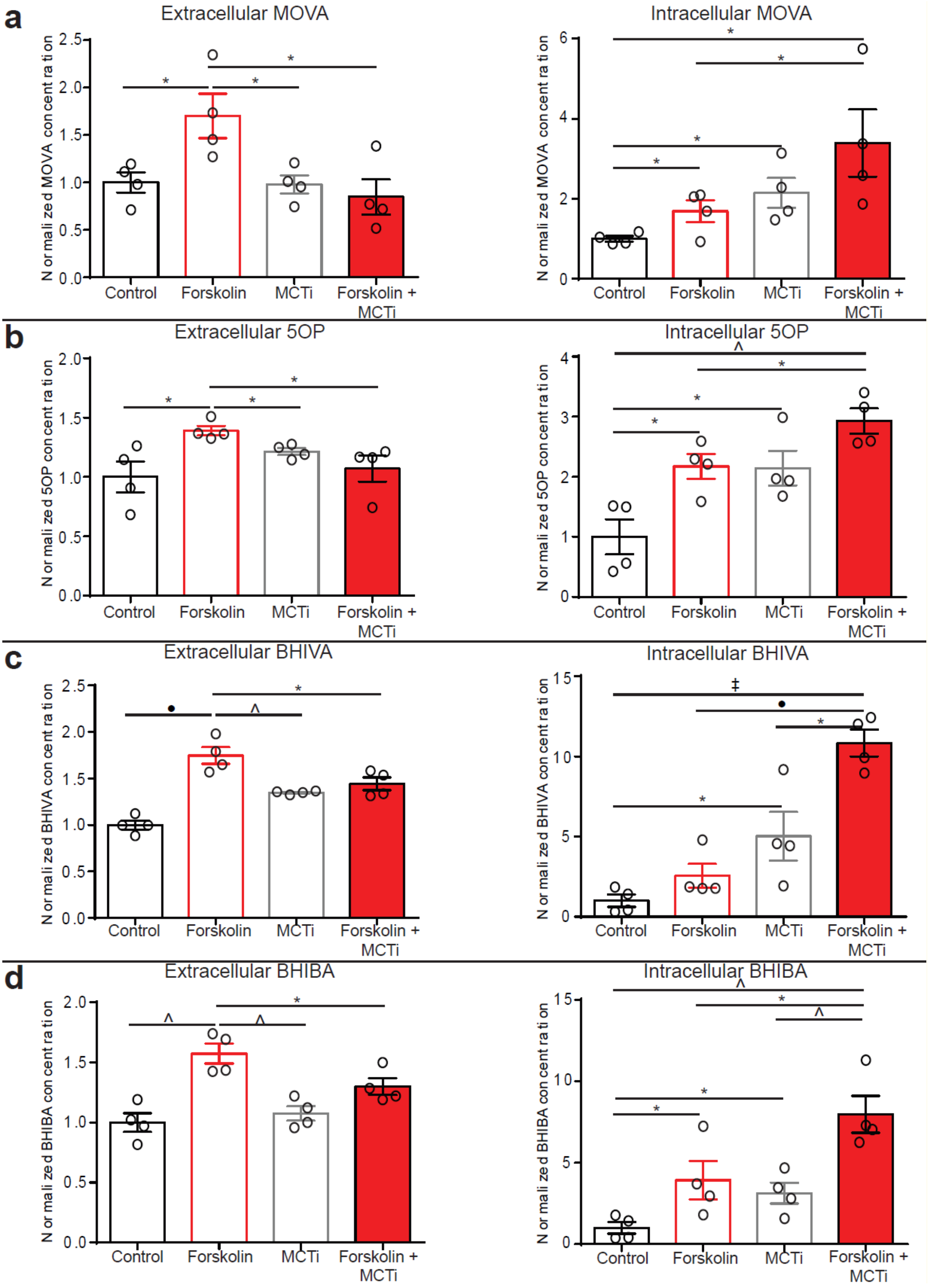

Supplementary Figure 4. Export of metabolite signals from browning adipocytes is mediated by monocarboxylate transporters

The concentration of (**a**) 3-methyl-2-oxovaleric acid (MOVA) (Extracellular; Control vs Forskolin p = 0.035, Forskolin vs MCTi p = 0.029, Forskolin vs Forskolin + MCTi p = 0.03) (Intracellular; Control vs Forskolin p = 0.047, Control vs MCTi p = 0.023, Control vs Forskolin + MCTi p = 0.03, Forskolin vs Forskolin + MCTi p = 0.027) (**b**) 5-oxoproline (5OP) (Extracellular; Control vs Forskolin p = 0.028, Forskolin vs MCTi p = 0.012, Forskolin vs Forskolin + MCTi p = 0.034) (Intracellular; Control vs Forskolin p = 0.018, Control vs MCTi p = 0.032, Control vs Forskolin + MCTi p = 0.0017, Forskolin vs Forskolin + MCTi p = 0.043) (**c**) β-hydroxyisovaleric acid (BHIVA) (Extracellular; Control vs Forskolin p = 0.0003, Forskolin vs MCTi p = 0.0045, Forskolin vs Forskolin + MCTi p = 0.036) (Intracellular; Control vs MCTi p = 0.042, Control vs Forskolin + MCTi p < 0.0001, Forskolin vs Forskolin + MCTi p = 0.003, MCTi vs Forskolin + MCTi p = 0.015) and (**d**) β–hydroxisobutyric acid (BHIBA) (Extracellular; Control vs Forskolin p = 0.0023, Forskolin vs MCTi p = 0.0028, Forskolin vs Forskolin + MCTi p = 0.04) (Intracellular; Control vs Forskolin p = 0.05, Control vs MCTi p = 0.028, Control vs Forskolin + MCTi p = 0.001, Forskolin vs Forskolin + MCTi p = 0.048, MCTi vs Forskolin + MCTi p = 0.0099) in adipocytes (intracellular) and media (extracellular) of cells treated with either forskolin (1 μM), the monocarboxylate transporter inhibitor (MCTi) α-cyano-4-hydroxycinnamate (2 mM) or both forskolin and α-cyano-4-hydroxycinnamate (n = 4; two-tailed t-test). ∗p ≤ 0.05, ^p ≤ 0.01, •p ≤ 0.001, ‡p ≤ 0.0001. Data are mean ± SEM with data points shown. Source data are provided as a Source Data file.

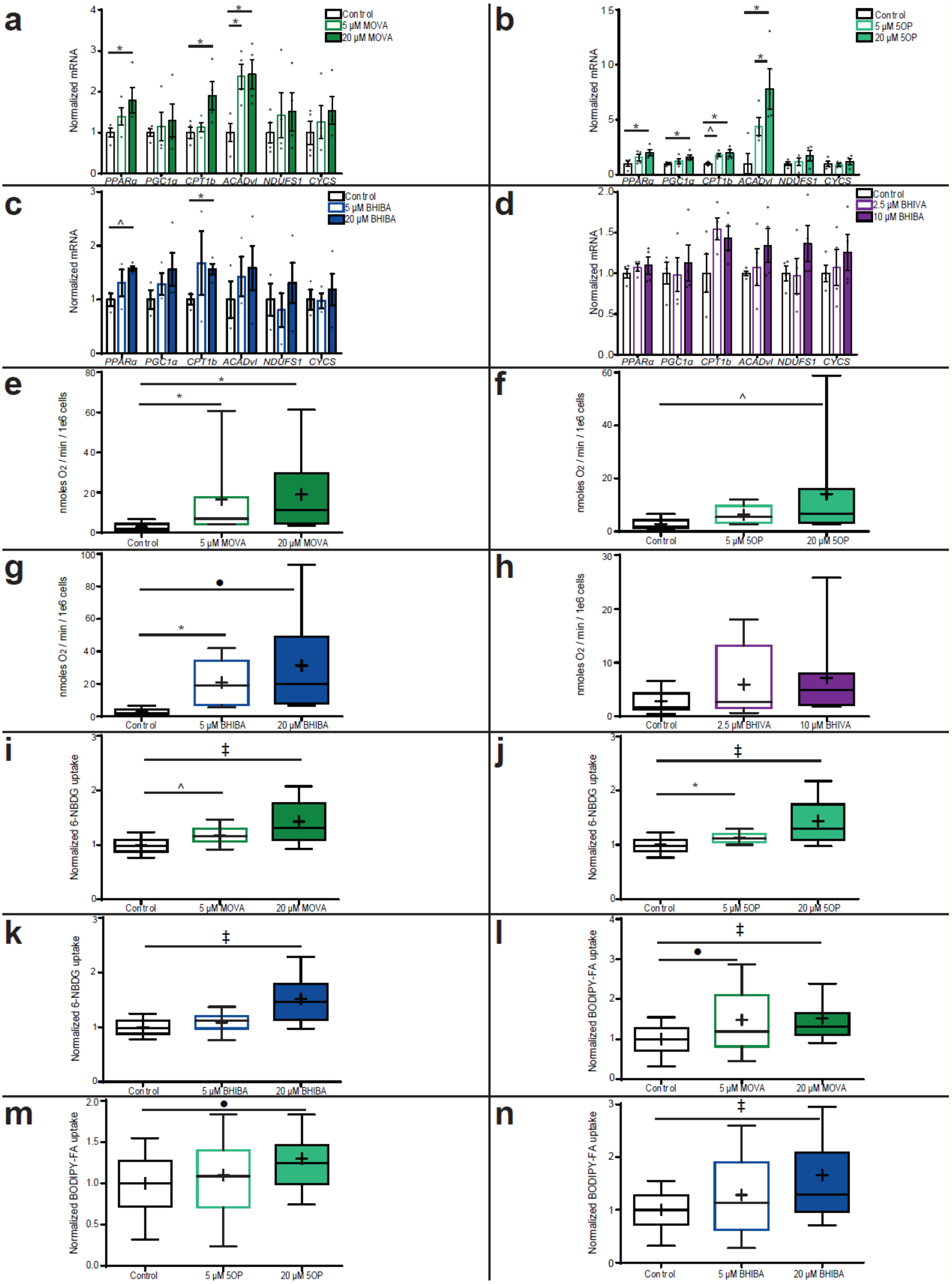

Supplementary Figure 5. MOVA, 5OP and BHIBA induce a dose responsive increase in oxidative energy metabolism in human primary skeletal myocytes

The expression of a mitochondrial and metabolic gene panel in human primary skeletal myocytes treated with (**a**) 3-methyl-2-oxovaleric acid (MOVA; 5 μM, 20 μM; n = 4; two-tailed t-test; 5 μM *ACADvl* $p = 0.011$; 20 μM PPARα $p = 0.05$, *CPT1b* $p = 0.05$, *ACADvl* $p = 0.015$), (**b**) 5-oxoproline (5OP; 5 μM, 20 μM; n = 4; two-tailed t-test; 5 μM *CPT1b* $p = 0.005$, *ACADvl* $p = 0.034$; 20 μM PPARα $p = 0.03$, *PGC1α* $p = 0.05$, *CPT1b* $p = 0.018$, *ACADvl* $p = 0.016$), (**c**) β-hydroxyisobutyric acid (BHIBA; Control n = 3, 5 μM n = 3, 20 μM n = 4; two-tailed t-test; 20 μM PPARα $p = 0.003$, *CPT1b* $p = 0.011$) (**d**) β-hydroxyisovaleric acid (BHIVA; 2.5 μM, 10 μM; n = 4). Basal oxygen consumption in human primary skeletal myocytes treated with (**e**) MOVA (Control n = 21; 5 and 20 μM n = 7; One-way ANOVA with Dunnett's post hoc; 5 μM $p = 0.034$, 20 μM $p = 0.012$) (**f**) 5OP (Control n = 21; 5 μM n = 7 and 20 μM n = 8; One-way ANOVA with Dunnett's post hoc; 20 μM $p = 0.0085$), (**g**) BHIBA (Control n = 21; 5 μM n = 6 and 20 μM n = 8; One-way ANOVA with Dunnett's post hoc; 5 μM $p = 0.03$, 20 μM $p = 0.0002$) and (**h**) BHIVA (Control n = 21; 2.5 μM n = 7 and 10 μM n = 8). Glucose uptake (6-(N-(7-Nitrobenz-2-oxa-1,3-diazol-4-yl)amino)-6-Deoxyglucose; 6-NBDG) in (**i**) MOVA (Control n = 180; 5 μM n = 36 and 20 μM n = 95; One-way ANOVA with Dunnett's post hoc; 5 μM $p = 0.0014$, 20 μM $p < 0.0001$), (**j**) 5OP (Control n = 180; 5 μM n = 36 and 20 μM n = 98; One-way ANOVA with Dunnett's post hoc; 5 μM $p = 0.02$, 20 μM $p < 0.0001$), (**k**) BHIBA (Control n = 180; 5 μM n = 36 and 20 μM n = 106; One-way ANOVA with Dunnett's post hoc; 20 μM $p < 0.0001$) treated human skeletal myocytes. Fatty acid (BODIPY-FA) uptake in (**l**) MOVA (Control n = 272; 5 μM n = 48 and 20 μM n = 144; One-way ANOVA with Dunnett's post hoc; 5 μM $p = 0.0001$, 20 μM $p < 0.0001$), (**m**) 5OP (Control n = 272; 5 μM n = 48 and 20 μM n = 144; One-way ANOVA with Dunnett's post hoc; 20 μM $p = 0.0001$), (**n**) BHIBA (Control n = 272; 5 μM n = 44 and 20 μM n = 140; One-way ANOVA with Dunnett's post hoc; 20 μM $p = 0.0001$) treated human skeletal myocytes. Dark green = MOVA, light green = 5OP, purple = BHIVA, dark blue = BHIBA. ∗p ≤ 0.05, ^p ≤ 0.01, •p ≤ 0.001, ‡p ≤ 0.0001. Data in bar charts are mean ± SEM with data points shown. Box and whisker plots show 25th to 75th percentile (box) min to max (whiskers), mean (+) and median (-). Source data are provided as a Source Data file.

.

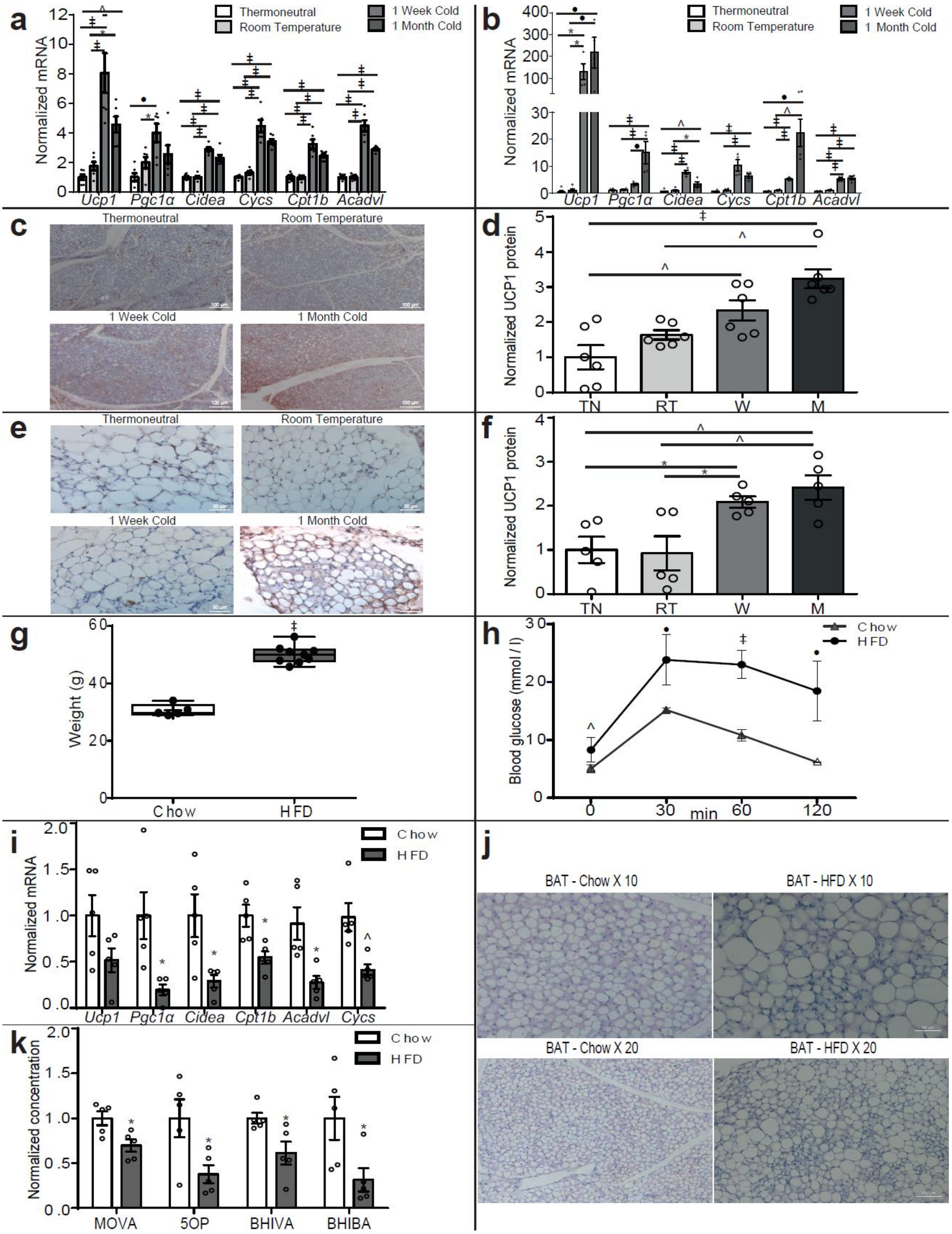

Supplementary Figure 6. Cold exposure induces a thermogenic phenotype and diet-induced obesity induces whitening to modulate metabokine concentrations in brown adipose tissue in mice

Thermogenic gene expression in the (**a**) interscapular brown adipose tissue (n = 6; One-Way ANOVA with Dunnett's post hoc; *Ucp1* TN vs W $p < 0.0001$, TN vs M $p = 0.008$, RT vs W $p < 0.0001$, RT vs M $p = 0.037$; *Pgc1α* TN vs W $p = 0.001$, RT vs W $p = 0.024$; *Cidea* TN vs W $p < 0.0001$, TN vs M $p < 0.0001$, RT vs W $p < 0.0001$, RT vs M $p < 0.0001$; *Cycs* TN vs W $p < 0.0001$, TN vs M $p < 0.0001$, RT vs W $p < 0.0001$, RT vs M $p < 0.0001$; *Cpt1b* TN vs W $p < 0.0001$, TN vs M $p < 0.0001$, RT vs W $p < 0.0001$, RT vs M $p < 0.0001$, *Acadvl* TN vs W $p < 0.0001$, TN vs M $p < 0.0001$, RT vs W $p < 0.0001$, RT vs M $p < 0.0001$) and (**b**) subcutaneous inguinal adipose tissue (TN, RT n = 6, W, M n = 4; One-Way ANOVA with Dunnett's post hoc; *Ucp1* TN vs W $p = 0.026$, TN vs M $p = 0.0005$, RT vs W $p = 0.026$, RT vs M $p = 0.0005$; *Pgc1α* TN vs M $p < 0.0001$, RT vs M $p < 0.0001$, W vs M $p = 0.0007$; *Cidea* TN vs W $p < 0.0001$, TN vs M $p = 0.0039$, RT vs W $p < 0.0001$, RT vs M $p = 0.014$; *Cycs* TN vs M $p < 0.0001$, RT vs M $p < 0.0001$; *Cpt1b* TN vs W $p < 0.0001$, TN vs M $p = 0.0008$, RT vs W $p < 0.0001$, RT vs M $p = 0.0016$; *Acadvl* TN vs W $p < 0.0001$, TN vs M $p < 0.0001$, RT vs W $p < 0.0001$, RT vs M $p < 0.0001$) of mice housed at thermoneutrality (TN), room temperature (RT), 8˚C for 1 week (W) or 8˚C for 1 month (M). (**c**) Immunohistochemical staining of UCP1 protein (red) counterstained with hematoxylin in the brown adipose tissue of cold conditioned mice (representative images of 6 independent repeats). (**d**) UCP1 protein is increased in the brown adipose tissue of cold conditioned mice (n = 6; One-Way ANOVA with Dunnett's post hoc; TN vs W, $p = 0.0064$, TN vs M $p < 0.0001$, RT vs M $p = 0.0013$). (**e**) Immunohistochemical staining of UCP1 protein (red) counter stained with hematoxylin in the subcutaneous adipose tissue of cold conditioned mice (representative images of 5 independent repeats). (**f**) UCP1 protein is increased in the subcutaneous adipose tissue of cold conditioned mice (n = 5; One-Way ANOVA with Dunnett's post hoc; TN vs W, $p = 0.043$, TN vs M $p = 0.0084$, RT vs W $p = 0.03$ RT vs M $p = 0.0057$). (**g**) Weights of mice receiving standard chow (Chow) or 60% fat diet (HFD) for 17 weeks (Chow n = 5; HFD n = 9; two-tailed t-test; $p < 0.0001$). (**h**) Intraperitoneal glucose tolerance test in Chow and HFD-fed mice (Chow n = 5; HFD n = 9; Two-Way ANOVA $p < 0.0001$; two-tailed t-test; 0 min $p = 0.006$, 30 min $p = 0.0009$, 60 min $p < 0.0001$, 90 min $p = 0.0002$). (**i**) Reduced thermogenic gene expression in the brown adipose tissue of HFD-fed mice compared to chow-fed mice n = 5; two-tailed t-test; *Pgc1α* $p = 0.015$; *Cidea* $p = 0.019$; *Cpt1b* $p = 0.011$; *Acadvl* $p = 0.011$ *Cycs* p = 0.008). (**j**) Hematoxylin and eosin stained brown adipose tissue from HFD-fed mice demonstrates whitening compared with brown adipose tissue from chow-fed mice (representative images of 5 independent repeats). (**k**) Concentrations of 3-methyl-2-oxovaleric acid (MOVA), 5-oxoproline (5OP), β-hydroxyisovaleric acid (BHIVA) and β-hydroxyisobutyric acid (BHIBA) are lower in brown adipose tissue from HFD mice (n = 5; two-tailed t-test; MOVA $p = 0.019$; 5OP $p = 0.028$, BHIVA $p = 0.026$, BHIBA $p = 0.036$). ∗p ≤ 0.05, ^p ≤ 0.01, •p ≤ 0.001, ‡p ≤ 0.0001. Data in bar charts are mean ± SEM with data points shown. Box and whisker plots show 25th to 75th percentile

(box) min to max (whiskers), mean (+) and median (-). Source data are provided as a Source Data file.

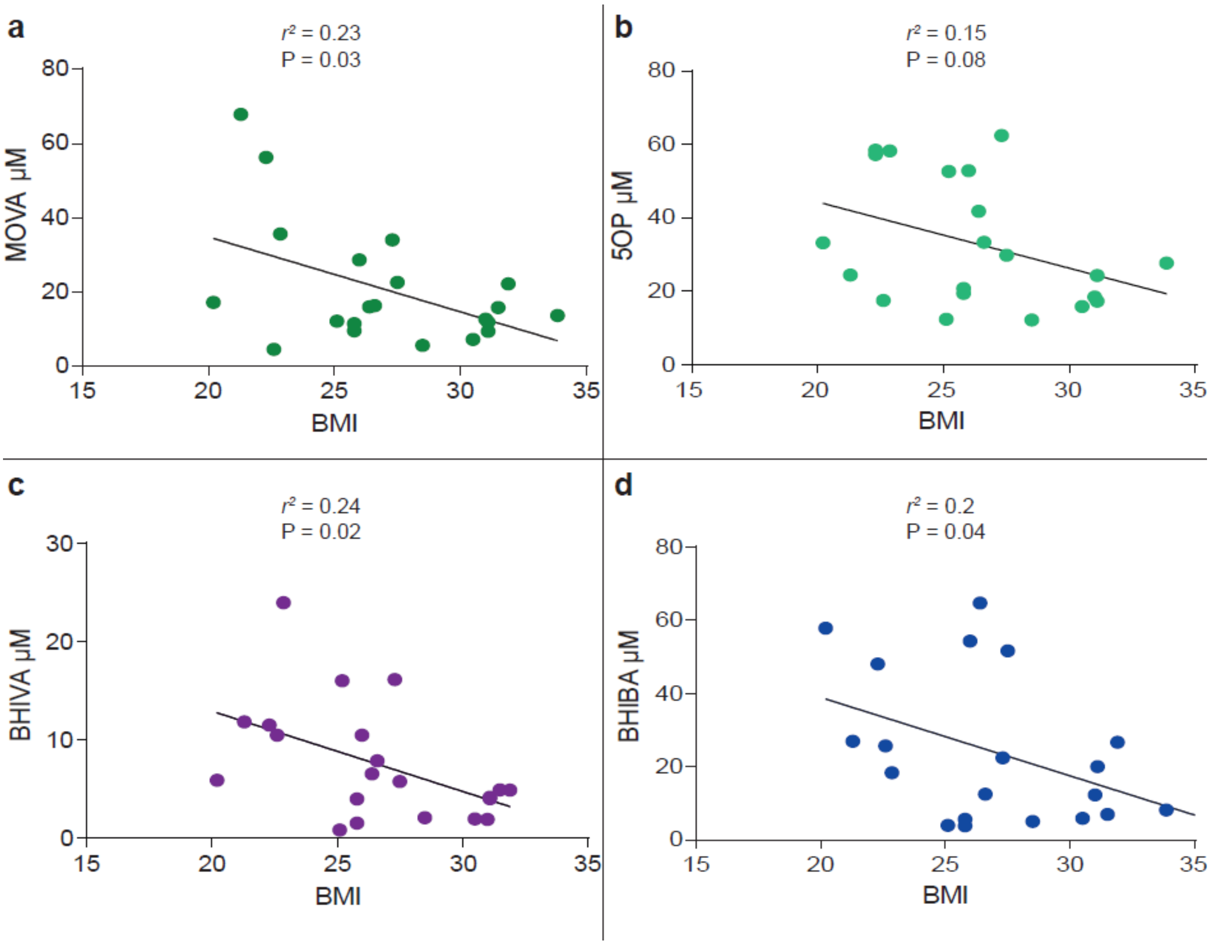

Supplementary Figure 7. Plasma concentrations of metabolites are inversely correlated with BMI in humans (a - d) The inverse correlation of 3-methyl-2-oxovaleric acid (MOVA) (r^2 = 0.2311, p = 0.027), 5-oxoproline (5OP) (r^2 = 0.1490, p = 0.084, β-hydroxyisovaleric acid (BHIVA) (r^2 = 0.2433, p = 0.023) and β-hydroxyisobutyric acid (BHIBA) (r^2 =0.2020, p = 0.041) plasma concentration to Body Mass Index (BMI) in human volunteers (n = 21). Dark green = MOVA, light green = 5OP, purple = BHIVA, dark blue = BHIBA. Analysis by Pearson correlation. Source data are provided as a Source Data file.

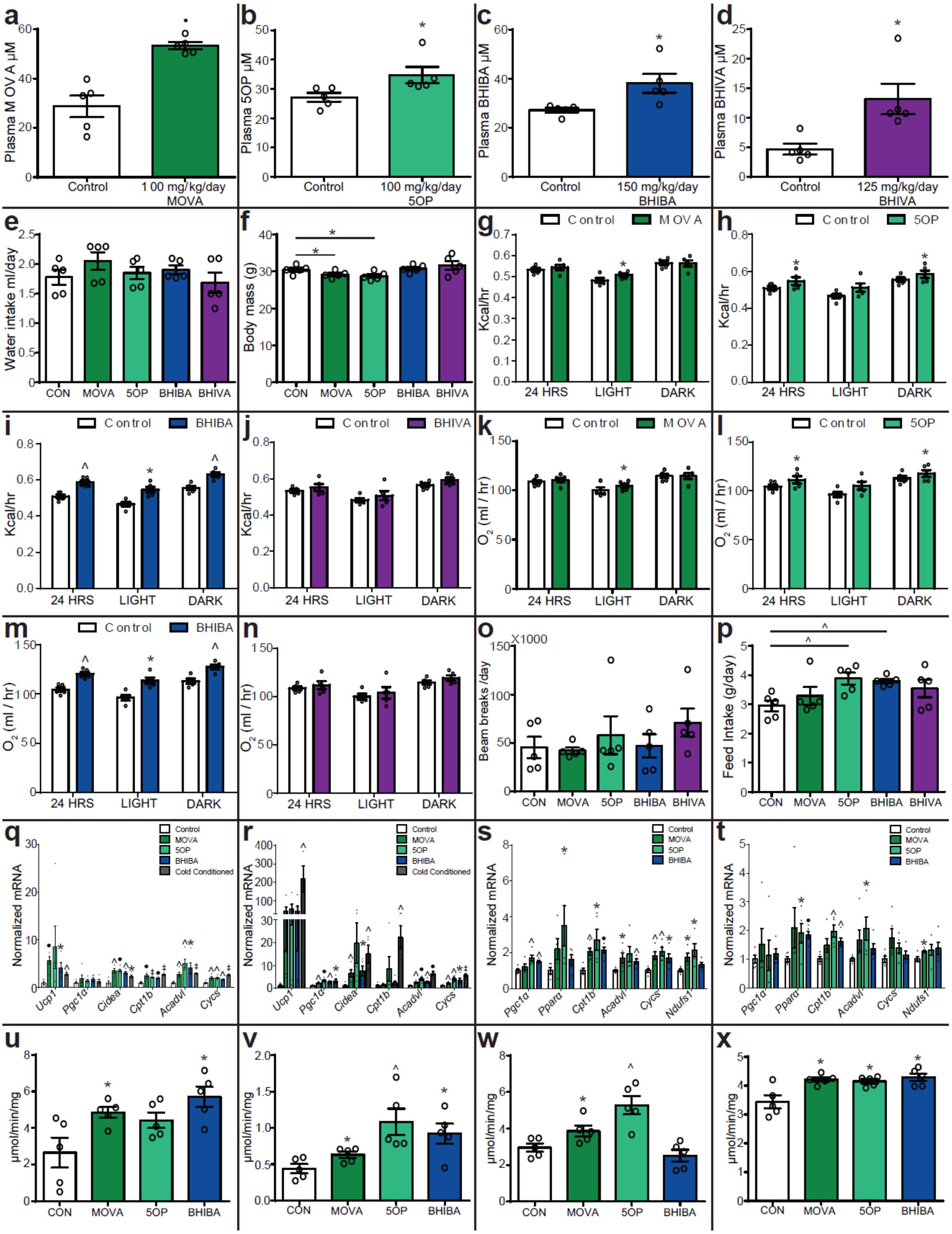

Supplementary Figure 8. Physiological concentrations of MOVA, 5OP and BHIBA increase systemic energy expenditure *in vivo*

(**a - d**) The plasma concentration of 3-methyl-2-oxovaleric acid (MOVA; p = 0.0007), 5-oxoproline (5OP; p = 0.045), β-hydroxyisobutyric acid (BHIBA; p = 0.025) and β-hydroxyisovaleric acid (BHIVA; p = 0.015) in mice given 100 mg/kg/day MOVA, 100 mg/kg/day

5OP, 150 mg/kg/day BHIBA and 125 mg/kg/day BHIVA, respectively, in their drinking water compared to control mice (n = 5; two-tailed t-test). (**e**) Water intake in MOVA, 5OP, BHIBA and BHIVA-treated mice (n = 5). (**f**) The weights of mice receiving MOVA, 5OP, BHIBA and BHIVA in their drinking water compared to untreated controls (n = 5; two-tailed t-test; MOVA $p = 0.05$, 5OP $p = 0.042$). Diurnal average energy expenditure for 24 hour period, 12 hour dark phase (DARK) and 12 hour light phase (LIGHT) of (**g**) MOVA (LIGHT $p = 0.027$) , (**h**) 5OP (24 hrs $p = 0.041$, DARK $p = 0.034$), (**i**) BHIBA (24 hrs $p = 0.008$, LIGHT $p = 0.025$, DARK $p = 0.009$) and (**j**) BHIVA treated mice (n = 5; ANCOVA with body mass as a covariate). Diurnal average oxygen consumption for 24 hours, 12 hour dark phase (DARK) and 12 hour light phase (LIGHT) of (**k**) MOVA (LIGHT $p = 0.05$), (**l**) 5OP (24 hrs $p = 0.035$, DARK $p = 0.031$), (**m**) BHIBA (24 hrs $p = 0.0066$, LIGHT $p = 0.024$, DARK $p = 0.0077$) and (**n**) BHIVA treated mice (n = 5; ANOCVA with body mass as a covariate). (**o**) Activity and (**p**) food consumption of MOVA, 5OP, BHIBA and BHIVA-treated mice (n = 5; two-tailed t-test; food consumption 5OP $p = 0.01$, BHIBA $p = 0.003$). Thermogenic and brown adipocyte-associated gene expression in (**q**) brown adipose tissue (BAT) (n = 5; two-tailed t-test; MOVA *Ucp1* $p = 0.0006$, *Cidea* $p = 0.003$, *Cpt1b* $p = 0.0007$, *Acadvl* $p = 0.00332334$, *Cycs* $p = 0.006$; 5OP *Cidea* $p = 0.0005$, *Cpt1b* $p < 0.0001$, *Acadvl* $p = 0.002$, *Cycs* $p = 0.0018$; BHIBA *Ucp1* $p = 0.019$, *Cidea* $p = 0.0014$, *Cpt1b* $p = 0.0005$, *Acadvl* $p = 0.025$, *Cycs* $p = 0.005$; Cold *Ucp1* $p = 0.003$, *Cidea* $p = 0.012$, *Cpt1b* $p < 0.0001$, *Acadvl* $p < 0.0001$, *Cycs* $p < 0.0001$) and (**r**) subcutaneous white adipose tissue (WAT) (n = 5, Cold Conditioned n = 4; two-tailed t-test; MOVA, *PGC1α* $p = 0.0017$, *Cidea* $p = 0.009$, *Acadvl* $p = 0.0014$, *Cycs* $p = 0.005$; 5OP *PGC1α* $p = 0.0004$, *Acadvl* $p = 0.0008$, *Cycs* $p = 0.0015$; BHIBA *PGC1α* $p = 0.0004$, *Cidea* $p = 0.02$, *Acadvl* $p = 0.0007$, *Cycs* $p = 0.05$; Cold *Ucp1* $p = 0.0097$, *PGC1α* $p = 0.027$, *Cidea* $p = 0.006$, *Cpt1b* $p = 0.002$, *Acadvl* $p = 0.00011$, *Cycs* $p < 0.0001$) of MOVA, 5OP, BHIBA and BHIVA-treated mice. Expression of mitochondrial and metabolic genes in the (**s**) gastrocnemius (MOVA *Cpt1b* $p = 0.0026$, *Acadvl* $p = 0.04$, *Cycs* $p = 0.007$, *Ndufs1* $p = 0.02$; 5OP *Pgc1α* $p = 0.007$, *Pparα* $p = 0.05$, *Cpt1b* $p = 0.02$, *Cycs* $p = 0.0028$, *Ndufs1* $p = 0.014$; BHIBA *Pgc1α* $p = 0.0019$, *Cpt1b* $p = 0.0007$, *Acadvl* $p = 0.007$, *Cycs* $p = 0.02$) and (**t**) soleus muscles (MOVA *Ndufs1* $p = 0.011$; 5OP *Pparα* $p = 0.034$, *Cpt1b* $p = 0.0027$, *Acadvl* $p = 0.03$; BHIBA *Pparα* $p = 0.0007$, *Cpt1b* $p = 0.005$) of metabokine-treated mice (n = 5; two-tailed t-test). Metabokine treatment increases mitochondrial content in (u) BAT (MOVA $p = 0.035$, BHIBA $p = 0.015$) (v) subcutaneous WAT (MOVA $p = 0.038$, 5OP $p = 0.01$, BHIBA $p = 0.013$) (w) gastrocnemius (MOVA $p = 0.046$, 5OP $p = 0.0029$) and (x) soleus muscle (MOVA $p = 0.012$, 5OP $p = 0.017$, BHIBA $p = 0.012$) of mice, determined using a citrate synthase assay (n = 5; two-tailed t-test). Dark green = MOVA, light green = 5OP, purple = BHIVA, dark blue = BHIBA. ∗p ≤ 0.05, ^p ≤ 0.01, •p ≤ 0.001, ‡p ≤ 0.0001. Data in bar charts are mean ± SEM with data points shown. Source data are provided as a Source Data file.

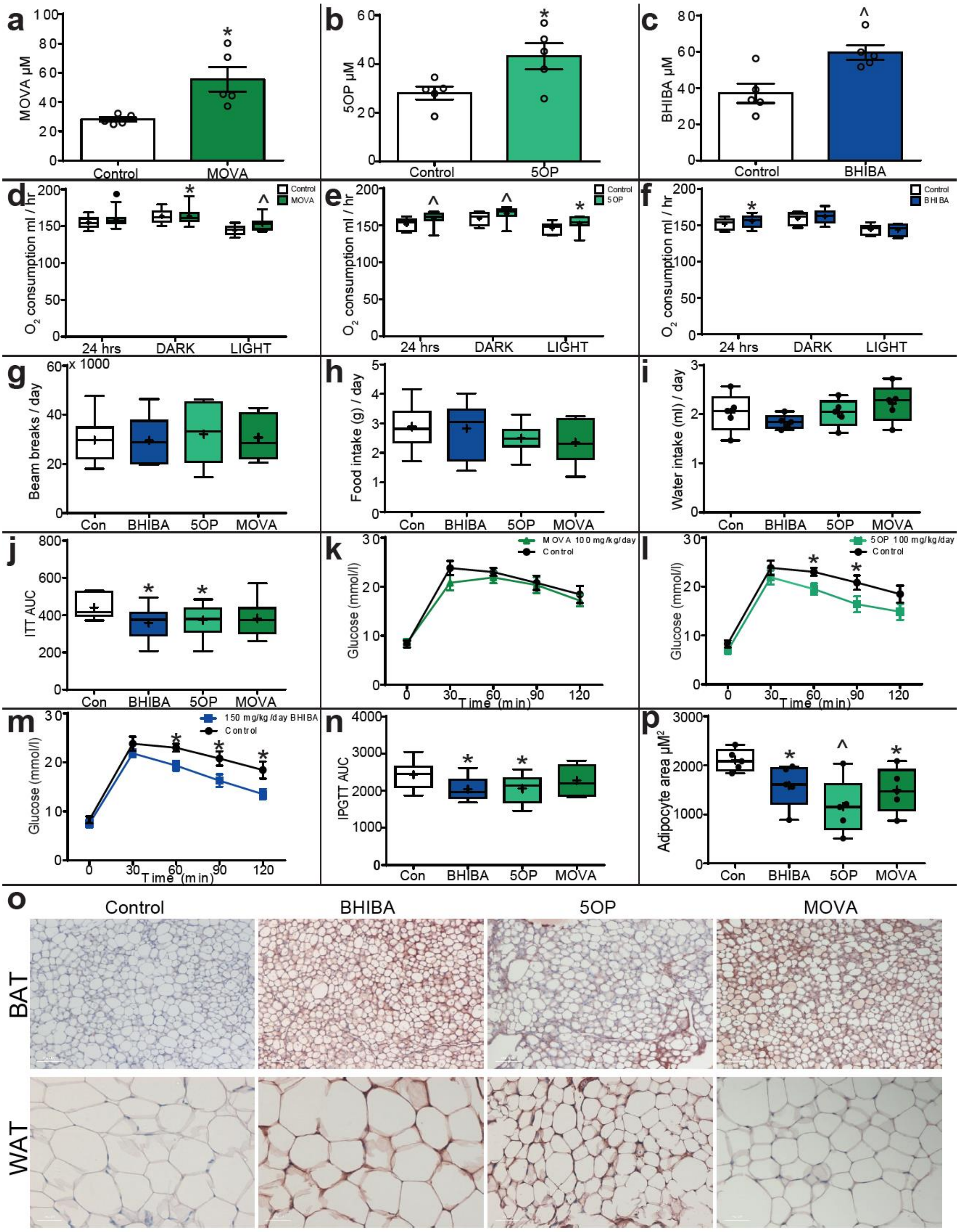

Supplementary Figure 9. The metabolites increase oxygen consumption, improve glucose tolerance and induce a phenotype and morphology consistent with browning in adipose tissue in obese mice

(**a - c**) The plasma concentration of 3-methyl-2-oxovaleric acid (MOVA) (p = 0.012, 5-oxoproline (5OP) (p = 0.035), and β-hydroxyisobutyric acid (BHIBA) (p = 0.01) in mice given

100 mg/kg/day MOVA, 100 mg/kg/day 5OP, or 150 mg/kg/day BHIBA, respectively, in their drinking water compared to control mice (n = 5; two-tailed t-test). Diurnal average oxygen consumption for 24 hour period, 12 hour dark phase (DARK) and 12 hour light phase (LIGHT) of (**d**) MOVA (n = 9; 24 hrs $p = 0.001$, DARK $p = 0.014$, LIGHT $p = 0.0092$) (**e**) 5OP (n = 9; 24 hrs $p = 0.005$, DARK $p = 0.032$, LIGHT $p = 0.005$), and (**f**) BHIBA (n = 8; 24 hrs $p = 0.016$) treated mice fed a 60% fat diet compared to controls (n = 8; ANCOVA with body weight as a covariate). (**g**) Daily activity (beam breaks) (Control, BHIBA n = 8; 5OP, MOVA n = 9), (**h**) food consumption (Control, BHIBA n = 8; 5OP, MOVA n = 9) and (**i**) water intake (n = 5) of mice treated with 100 mg/kg/day MOVA, 100 mg/kg/day 5OP or 150 mg/kg/day BHIBA. (**j**) Area under the curve (AUC) of intraperitoneal insulin tolerance tests of BHIBA, 5OP, or MOVA-treated mice (n = 10, Control n = 9; two-tailed t-test; BHIBA $p = 0.019$, 5OP $p = 0.043$). Intraperitoneal glucose tolerance tests of (**k**) MOVA, (**l**) 5OP (Two-Way ANOVA $p = 0.0006$; two-tailed t-test, 60 min $p = 0.02$, 90 min $p = 0.05$) and (**m**) BHIBA (Two-Way ANOVA $p < 0.0001$; two-tailed t-test, 60 min $p = 0.012$, 90 min $p = 0.03$, 120 min $p = 0.027$) treated mice (n = 9). (**n**) AUC of intraperitoneal glucose tolerance tests of BHIBA ($p = 0.024$), 5OP ($p = 0.045$), or MOVA-treated mice. (n = 9; two-tailed t-test) (**o**) Immunohistochemical staining of Ucp1 protein (red) counterstained with hematoxylin in the brown adipose tissue (top panels) and inguinal subcutaneous adipose tissue (bottom panels) of BHIBA, 5OP and MOVA-treated mice (representative images of 5 independent repeats). (**p**) Adipocyte area of the subcutaneous adipose tissue of BHIBA ($p = 0.046$), 5OP ($p = 0.008$), or MOVA ($p = 0.027$) treated mice with data points shown (n = 5; two-tailed t-test). Dark green = MOVA, light green = 5OP, dark blue = BHIBA. ∗p ≤ 0.05, ^p ≤ 0.01, •p ≤ 0.001, ‡p ≤ 0.0001. Data in bar charts are mean ± SEM with data points shown. Box and whisker plots show 25th to 75th percentile (box) min to max (whiskers), mean (+) and median (-). Source data are provided as a Source Data file.

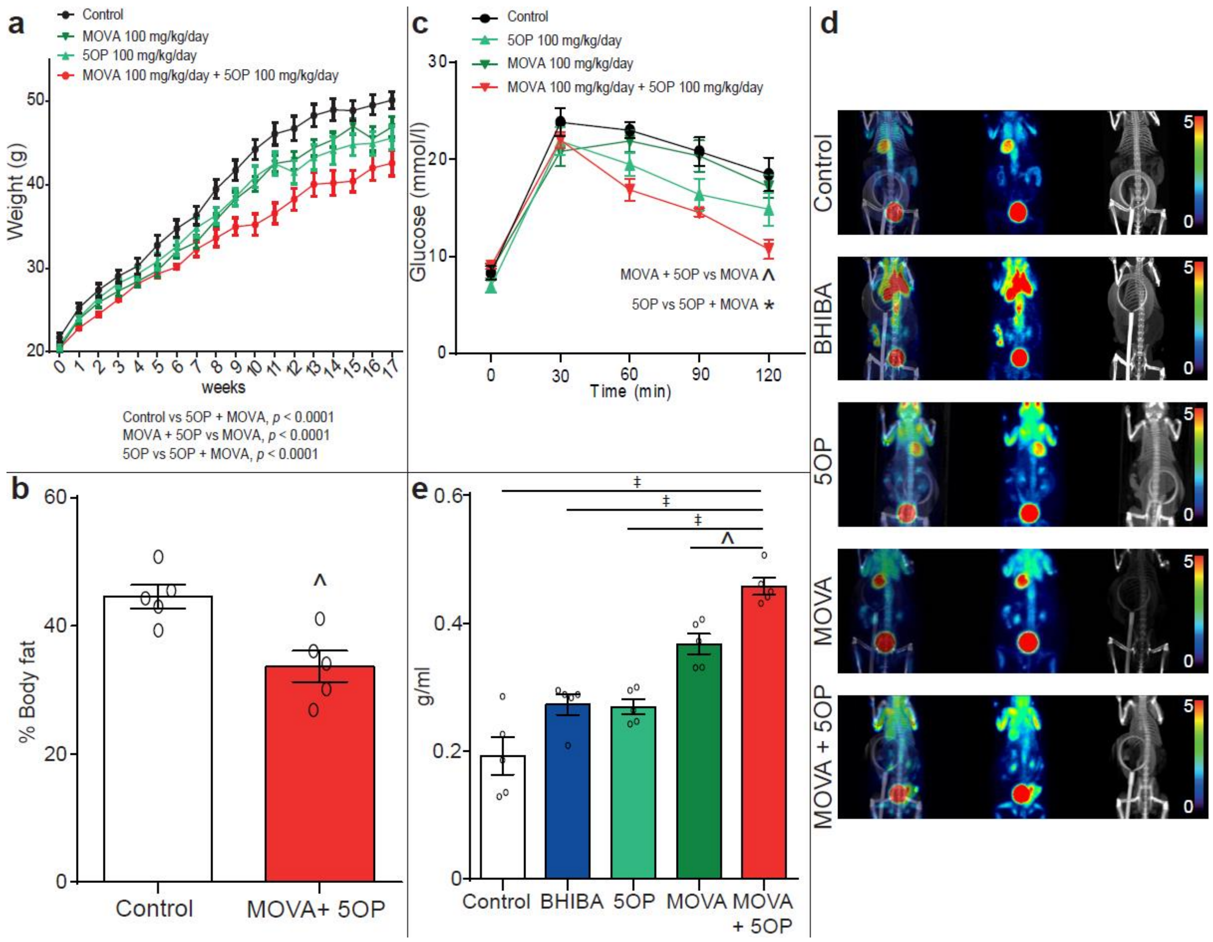

Supplementary Figure 10. The metabolites MOVA and 5OP have additive effects on body weight and glucose disposal in high fat diet-fed mice

(**a**) The reduction in weight gain in mice given a combination of 3-methyl-2-oxovaleric acid (MOVA) and 5-oxoproline (5OP) was greater than either MOVA or 5OP treatments (Control n = 9; MOVA, 5OP n = 10; MOVA + 5OP n = 5; Two-way ANOVA with Holm-Sidak post hoc; Control vs MOVA + 5OP $p < 0.0001$; MOVA vs MOVA + 5OP $p < 0.0001$; 5OP vs 5OP + MOVA $p < 0.0001$). (**b**) Computed Tomography demonstrates that combined MOVA and 5OP treatment significantly reduces adiposity in mice (n = 5; two-tailed t-test; $p = 0.007$). (**c**) Intraperitoneal glucose tolerance tests showed a greater improvement in glucose tolerance in the MOVA and 5OP combination-treated mice (Control; MOVA, 5OP n = 9; MOVA + 5OP n = 5; Two-Way ANOVA with Holm-Sidak post hoc; 120 min; MOVA vs MOVA + 5OP $p = 0.004$, 5OP vs MOVA + 5OP $p = 0.046$). (**d**) Positron emission tomography / computed tomography (PET/CT) ^{18}F-fluorodeoxyglucose (^{18}F-FDG) imaging of BHIBA, 5OP, MOVA and a combination of MOVA and 5OP-treated mice identifies enhanced glucose uptake of the combined treatment *in vivo* (Representative images from 5 independent repeats). Scale bars are standardized uptake value (SUV) in rainbow scale (0 violet – 5 red). Right – CT, middle - PET, left – PET/CT co-registration. (**e**) PET/CT demonstrates that a combined MOVA and

5OP treatment significantly increases uptake of the glucose analogue ^{18}F-FDG into the skeletal muscle of the hind limbs compared with either 5OP or MOVA treatment alone, expressed as standardized uptake values g/ml ($n = 5$; Two-Way ANOVA with Holm-Sidak post hoc; Control vs 5OP + MOVA $p < 0.0001$, BHIBA vs 5OP + MOVA $p < 0.0001$, 5OP vs 5OP + MOVA $p < 0.0001$, MOVA vs 5OP + MOVA $p = 0.0024$). Dark green = MOVA, light green = 5OP, dark blue = BHIBA, red = MOVA + 5OP. ∗$p \leq 0.05$, ^$p \leq 0.01$, •$p \leq 0.001$. Data in bar charts are mean ± SEM with data points shown. Source data are provided as a Source Data file.

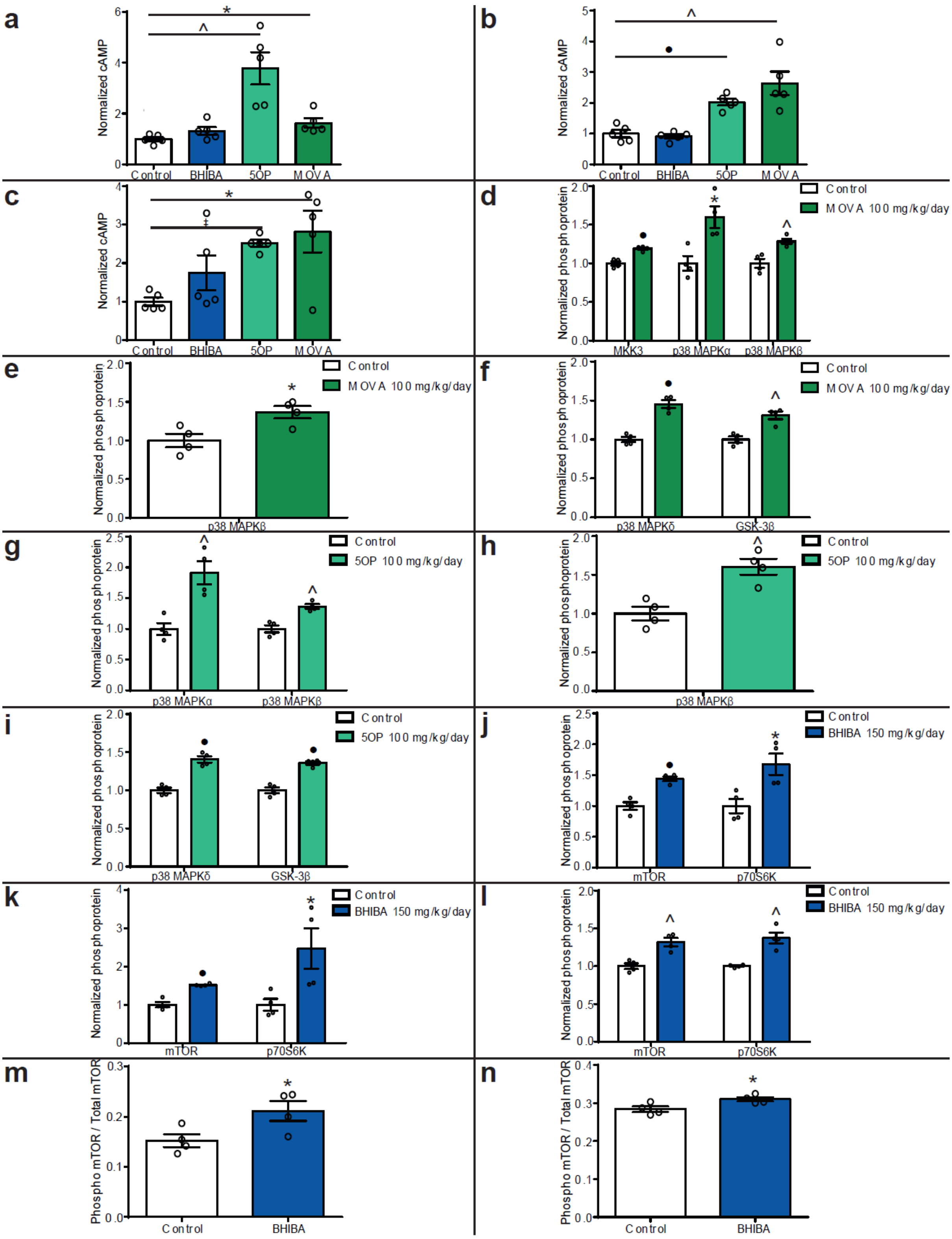

Supplementary Figure 11. MOVA and 5OP signal through cAMP-PKA-p38 MAPK and BHIBA via mTOR to regulate adipocyte and myocyte metabolic gene expression

Cyclic AMP concentrations in the (**a**) brown adipose tissue (BAT) (5OP p = 0.0024, MOVA p = 0.014), (**b**) inguinal white adipose tissue (WAT) (5OP p = 0.0002, MOVA p = 0.0034) and (**c**) soleus skeletal muscle (5OP p < 0.0001, MOVA p = 0.011 of mice treated with 100

mg/Kg/day 3-methyl-2-oxovaleric acid (MOVA), 100 mg/Kg/day 5-oxoproline (5OP) or 125 mg/Kg/day β-hydroxyisobutyric acid (BHIBA) for 17 weeks (n = 5). MOVA increased (**d**) brown adipose tissue (BAT) MKK3 (p = 0.0009), p38 MAPKα (p = 0.012) and p38MAPKβ (p = 0.0057), (**e**) inguinal white adipose tissue (WAT) p38MAPKβ (p = 0.02) and (**f**) soleus p38 MAPKδ (p = 0.0003) and GSK3β (p = 0.0026) phosphorylation in mice (n = 4). 5OP increased (**g**) BAT p38MAPKα (p = 0.0048) and p38MAPKβ (p = 0.0019), and (**h**) inguinal WAT p38MAPKβ (p = 0.0042) and (**i**) soleus p38MAPKδ (p = 0.00029) and GSK3β (p = 0.00019) phosphorylation in mice (n = 4). BHIBA increased phosphorylation of mTOR and p70S6K in (**j**) BAT (mTOR p = 0.0008; p70S6K p = 0.018), (**k**) inguinal WAT (mTOR p = 0.0003; p70S6K p = 0.038) and (**l**) soleus muscle (mTOR p = 0.003; p70S6K p = 0.002) of mice (n = 4). The phospho mTOR / total mTOR ratio in (**m**) BAT (p = 0.046) and (**n**) soleus skeletal muscle (p = 0.03) of 125 mg/Kg/day BHIBA treated mice. Dark green = MOVA, light green = 5OP, dark blue = BHIBA. Significance was derived from a two-tailed t-test. ∗p ≤ 0.05, ^p ≤ 0.01, •p ≤ 0.001, ‡p ≤ 0.0001. Data are mean ± SEM with individual data points shown. Source data are provided as a Source Data file.

Metabolite	Plasma Concentration (μM)	Reference
α-hydroxyisocaproic acid (HIC)	0.25 ± 0.02	Hoffer et al.[1]
α-ketoisovaleric acid (AKV)	11 ± 1.7<br><br>14 (0 – 28)	Geigy Scientific Tables, 8th Rev edition, pp. 165-177.<br><br>Hoffmann et al.[2]
α-hydroxyisovaleric acid (AHI)	4.5 (2.9-6.2)<br><br>7.7 (0.0-19.0)	Geigy Scientific Tables, 8th Rev edition, pp. 165-177.<br><br>Hoffmann et al.[2]
3-methyl-2-oxovaleric acid (MOVA)	22.7 +/- 4.6<br><br>18.0 (8.0-31.0)	Geigy Scientific Tables, 8th Rev edition, pp. 165-177.<br><br>Hoffmann et al.[2]
β-hydroxyisobutyric acid (BHIBA)	21.0 +/- 2.0	Avogaro et al[3]
β-hydroxyisovaleric acid (BHIVA)	≤10	Engelke et al.[4]
5-oxo-proline (5OP)	19.5 ± 3.7	Friesen et al.[5]

Supplementary Table 1. Physiological concentration of candidate metabokines in human plasma.

Target	Fold-change	*p*-value
UCP1	20.754	0.06
LPL	6.61	0.06
LEP	5.985	0.02
CKMT2	4.028	0.06
FASN	3.252	0.03
NPR1	3.185	0.04
SLC36A2	3.1	0.06
ADIPOQ	3.04	0.03
CEBPA	3.017	0.04
IL1B	2.755	0.08
NR1H3	2.622	0.01
PNPLA2	2.559	0.06
FABP4	2.311	0.01
CKMT1B	2.271	0.009
Perilipin 1	2.199	0.04
MLXIPL	2.099	0.05
CPT1B	2.065	0.09
GATM	2.03	0.07
SLC27A1	1.908	0.02
SLC2A4	1.894	0.02
CD36	1.801	0.03
PGC1A	1.738	0.01
SPARC	1.655	0.001
Adipsin	1.623	0.006
PRDM16	1.611	0.007
NPR3	1.539	0.04
PPARG	1.49	0.05
INSR	1.436	0.02
PPARA	1.383	0.03
SREBF1	1.297	0.06
BMPR1A	1.286	0.04
IGF1R	1.258	0.03
ELOVL6	1.247	0.01
ATF2	1.234	0.03
CREB1	1.207	0.05
NRF1	1.158	0.03

Supplementary Table 2. Table of gene expression array data of key adipocyte and brown adipocyte-associated genes changed in immortalized human white preadipocytes isolated from neck fat and differentiation to adipocytes in the presence of 3-methyl-2-oxovaleric acid (20 μM) (Expression targets ordered by magnitude fold change, $p \leq 0.1$; Benjamini Hochberg-adjusted two-tailed t-tests; n = 3). Source data are provided as a Source Data file.

Target	Fold-change	*p*-value
UCP1	14.776	0.08
LEP	5.731	0.02
CKMT1A/B	4.695	0.05
CKMT2	4.205	0.05
NPR1	3.677	0.02
FABP4	3.468	0.06
FASN	3.053	0.03
CEBPA	2.919	0.04
ADIPOQ	2.793	0.03
PNPLA2	2.606	0.06
NR1H3	2.477	0.01
MLXIPL	2.447	0.03
Perilipin 1	2.371	0.03
Adipsin	2.323	0.02
SLC27A1	2.09	0.02
CD36	2.054	0.07
SLC2A4	1.762	0.05
NPR3	1.629	0.03
INSR	1.497	0.07
SPARC	1.486	0.03
PPARG	1.482	0.05
SREBF1	1.431	0.003
NRF1	1.4	0.03
ELOVL6	1.317	0.02
ATF2	1.297	0.05
CREB1	1.295	0.08
BMPR1A	1.176	0.05

Supplementary Table 3. Table of gene expression array data of key adipocyte and brown adipocyte-associated genes changed in immortalized human white preadipocytes isolated from neck fat and differentiation to adipocytes in the presence of 5-oxo-proline (20 μM) (Expression targets ordered by magnitude fold change, $p \leq 0.1$; Benjamini Hochberg-adjusted two-tailed t-tests; n = 4). Source data are provided as a Source Data file.

Target	Fold change	*p*-value
LEP	3.484	0.06
ADRB3	3.327	0.08
Adipsin	2.204	0.01
PGC1A	2.019	0.009
SLC27A1	1.747	0.004
MLXIPL	1.599	0.08
INSR	1.58	0.006
PRDM16	1.546	0.01
IGF1R	1.493	0.004
NPR3	1.489	0.04
NRF1	1.476	0.002
SPARC	1.473	0.01
CEBPA	1.455	0.09
BMPR1A	1.327	0.01
PPARG	1.324	0.09
ATF2	1.292	0.01
TFAM	1.291	0.08
CREB1	1.258	0.02
COL6A3	1.191	0.06
ELOVL6	1.106	0.07
NRF1	1.077	0.07
FOXC2	0.753	0.03
CIDEA	0.119	0.09

Supplementary Table 4. Table of gene expression array data of key adipocyte and brown adipocyte-associated genes changed in immortalized human white preadipocytes isolated from neck fat and differentiation to adipocytes in the presence of β-hydroxyisobutyric acid (20 μM) (Expression targets ordered by magnitude fold change, $p \leq 0.1$; Benjamini Hochberg-adjusted two-tailed t-tests; n = 4). Source data are provided as a Source Data file.

Target	Fold-change	*p*-value
UCP1	21.663	0.06
CKMT1A/B	4.921	0.05
LPL	4.712	0.09
CKMT2	3.214	0.09
FABP4	3.213	0.07
LEP	2.862	0.09
SLC36A2	2.655	0.08
NPR1	2.622	0.07
IL1B	2.599	0.09
CEBPA	2.555	0.06
ADIPOQ	2.438	0.06
NR1H3	2.392	0.02
PNPLA2	2.222	0.09
CD36	2.11	0.06
FASN	2.085	0.09
Perilipin 1	1.995	0.05
MLXIPL	1.954	0.06
CPT1B	1.917	0.04
Adipsin	1.884	0.007
SLC2A4	1.626	0.06
NPR3	1.596	0.02
SPARC	1.551	0.002
PPARG	1.455	0.06
INSR	1.439	0.002
BMPR1A	1.407	0.009
SREBF1	1.294	0.03
ELOVL6	1.244	0.007

Supplementary Table 5. Table of gene expression array data of key adipocyte and brown adipocyte-associated genes changed in immortalized human white preadipocytes isolated from neck fat and differentiation to adipocytes in the presence of β-hydroxyisovaleric acid (10 μM) (Expression targets ordered by magnitude fold change, $p \leq 0.1$; Benjamini Hochberg-adjusted two-tailed t-tests; n = 4). Source data are provided as a Source Data file.

Variant ID	Gene	SNP ID	*p*-Value	Effect	Trait	Data Set
6_80866657_T_G	*BCKDHB*	rs13220420	0.00000750	-0.0210	BMI	GIANT-UK Biobank GWAS Meta-analysis: males
6_80952806_A_G	*BCKDHB*	rs17506768	0.000149	-0.0763	BMI	GIANT GWAS: active men
6_81034583_G_A	*BCKDHB*	rs806848	0.000200	-0.00970	BMI	GIANT-UK Biobank GWAS Meta-analysis
6_81035173_A_G	*BCKDHB*	rs806845	0.000247	-0.00950	BMI	GIANT-UK Biobank GWAS Meta-analysis
6_81034730_A_G	*BCKDHB*	rs806847	0.000262	-0.00950	BMI	GIANT-UK Biobank GWAS Meta-analysis
6_80977740_G_T	*BCKDHB*	rs3805901	0.000300	-0.00980	BMI	GIANT UK Biobank GWAS
6_81036745_G_A	*BCKDHB*	rs1474790	0.000304	-0.00940	BMI	GIANT-UK Biobank GWAS Meta-analysis
6_81035737_C_A	*BCKDHB*	rs7747016	0.000307	-0.00940	BMI	GIANT-UK Biobank GWAS Meta-analysis
19_49299109_T_C	*BCAT2*	rs73587808	0.000488	0.255	BMI	GIANT-UK Biobank GWAS Meta-analysis: females
12_121167675_G_A	*ACADS*	rs12369156	0.000131	-0.0222	BMI	GIANT-UK Biobank GWAS Meta-analysis
12_121179939_C_T	*ACADS*	rs12825376	0.000137	0.0138	BMI	GIANT-UK Biobank GWAS Meta-analysis: females
12_121177120_A_G	*ACADS*	rs566325901	0.000383	0.167	BMI	GIANT-UK Biobank GWAS Meta-analysis
2_26472711_G_C	*HADHA*	rs559393527	0.0000341	-0.124	BMI	GIANT-UK Biobank GWAS Meta-analysis
2_26472712_A_T	*HADHA*	rs529693611	0.0000341	-0.124	BMI	GIANT-UK Biobank GWAS Meta-analysis
2_26472713_A_G	*HADHA*	rs548177580	0.0000371	-0.123	BMI	GIANT-UK Biobank GWAS Meta-analysis
2_26472714_T_C	*HADHA*	rs563090369	0.0000371	-0.123	BMI	GIANT-UK Biobank GWAS Meta-analysis

2_26479414_T_C	*HADHA*	rs397984129	0.000461	-0.0873	BMI	GIANT-UK Biobank GWAS Meta-analysis
20_33523155_G_T	*GSS*	rs2236270	3.60e-8	0.00980	BMI	GIANT UK Biobank GWAS
20_33525407_G_A	*GSS*	rs7265992	8.40e-7	-0.0113	BMI	GIANT UK Biobank GWAS
20_33547633_T_G	*GSS*	rs6088662	0.0000250	0.00930	BMI	GIANT UK Biobank GWAS
20_33545055_G_A	*GSS*	rs13041792	0.0000260	0.00930	BMI	GIANT UK Biobank GWAS
20_33529766_T_G	*GSS*	rs2273684	0.0000280	0.00690	BMI	GIANT UK Biobank GWAS
20_33542605_T_C	*GSS*	rs6088659	0.0000285	0.0328	BMI	GIANT GWAS: men, Europeans, active + inactive individuals
20_33538214_G_A	*GSS*	rs35416056	0.0000326	0.0175	BMI	GIANT-UK Biobank GWAS Meta-analysis
20_33522054_T_C	*GSS*	rs6087653	0.0000429	0.0250	BMI	GIANT GWAS: men, active + inactive individuals
20_33540000_G_A	*GSS*	rs2025096	0.0000580	0.00890	BMI	GIANT UK Biobank GWAS
20_33527838_A_G	*GSS*	rs6088655	0.0000679	0.0248	BMI	GIANT GWAS: men, active + inactive individuals
20_33544075_C_G	*GSS*	rs3761144	0.0000719	0.0243	BMI	GIANT GWAS: men, active + inactive individuals
7_30544048_C_T	*GGCT*	rs549124813	0.0000875	0.174	BMI	GIANT-UK Biobank GWAS Meta-analysis: females
1_113504486_C_T	*SLC16A1*	rs186286251	0.000471	0.296	BMI	GIANT-UK Biobank GWAS Meta-analysis: males

Supplementary Table 6 Genetic variants in the metabokine biosynthetic genes associated with Body Mass Index in Genome Wide Association Study database in Genetic Investigation of ANthropometric Traits (GIANT) and UK Biobank Meta-analysis, including 795,640 subjects in the Type 2 Diabetes Knowledge Portal (http://www.type2diabetesgenetics.org/). *p*-value cut-off < 0.0005.

Volunteers	Age (years)	% Male	Weight (kg)	Mean body mass index
42	74.9 ± 1.3	78.6	84.5 ± 1.3	27.6 ± 0.7

Supplementary Table 7. Morphological parameters for human adipose biopsy volunteers. Data shown is Mean ± SEM.

	MOVA	5OP	BHIBA	BHIVA
**Mouse Primary Adipocytes**				
Secretion during browning	↑	↑	↑	↑
Induce brown adipocyte gene expression	↑↑	↑↑	↑	↑
**Human Primary Adipocytes**				
Secretion during browning	↑	↑	↑	↑
Brown adipocyte gene expression	↑	↑	↑	↑
Cellular respiration	↑↑	↑↑	↑	↑
UCP1 Protein expression	↑	↑	↑	−
Relative fatty acid oxidation	↑	↑	↑↑	−
Glucose uptake	−	↑	↑	↑
Fatty acid uptake	↑	↑	↑	↑
**Mouse C2C12 Myotubes**				
Metabolic gene expression	↑↑	↑↑	↑	↑
**Human Primary Skeletal Myotubes**				
Metabolic gene expression	↑↑	↑↑	↑	−
Cellular respiration	↑	↑	↑	−
Glucose uptake	↑	↑	−	
Fatty acid uptake	↑	↑	↑	
**High Fat Diet-fed C57Bl6 Mice**				
Weight gain	↓↓	↓↓	↓	−
Adiposity	↓	↓	−	
Energy expenditure	↑	↑	↑	
Oxygen consumption	↑	↑	↑	
Insulin sensitivity	↑	↑↑	↑↑	−
Glucose tolerance	−	↑	↑	−
BAT Mitochondrial content	−	↑	↑	
Subcutaneous WAT Mitochondrial content	−	↑	↑	
Soleus mitochondrial content	↑	↑	↑	
In vivo BAT glucose uptake	↑	−	↑↑	
In vivo hind limb glucose uptake	↑	↑	↑	
In vivo fore limb glucose uptake	↑	↑	−	

Supplementary Table 8. Summary of the key metabolic effects of 3-methyl-2-oxovaleric acid (MOVA), 5-oxo-proline (5OP), β-hydroxyisobutyric acid (BHIBA) and β-hydroxyisovaleric acid (BHIVA) in mouse and human primary adipocytes, mouse and human skeletal myotubes and C57Bl6/J mice *in vivo*.

Gene	ThermoFisher Assay ID	Primer Reference Sequence	Amplicon Length
NRF1	Hs00602161_m1	NM_001293163.1; NM_001040110.1; NM_005011.4; NM_001293164.1	80
TFAM	Hs01073349_g1	NR_073073.1; XM_011540120.2; NM_001270782.1; XM_011540121.2; NM_003201.2	64
PRDM16	Hs00223161_m1	XM_005244772.4; XM_005244773.4; XM_005244774.4; XM_017002050.1; XM_011541945.2; NM_022114.3; XM_006710814.3; NM_199454.2	68
ADRB3	Hs00609046_m1	NM_000025.2	65
CIDEA	Hs00154455_m1	NM_001318383.1; NM_001279.3; NR_134607.1	76
ATF2	Hs01095345_m1	NR_045774.1; NR_045772.1; NM_001256094.1; NM_001256090.1; NR_045771.1; NM_001256092.1; NR_045769.1; NR_045773.1; NM_001880.3; NR_045770.1; NM_001256091.1; NR_045768.1; NM_001256093.1	67
CREB1	Hs00231713_m1	NM_134442.4; XM_011510646.2; XM_017003400.1; XM_011510645.1; XM_017003399.1; NR_135473.1; XM_011510651.2; XM_011510649.2; XM_011510650.2; NM_001320793.1; XM_017003401.1; XM_011510647.2; XM_011510648.2; NM_004379.4	75
CKMT1B	Hs00179727_m1	NM_020990.4; XM_017021902.1; XM_011521198.1;	85

		XM_011521197.2;XM_005254150.3; XM_011521199.2; NM_001321926.1; NM_001321927.1; NR_135856.1; NM_001015001.2; NM_001321928.1; NM_001321929.1; XM_011521194.1; XM_011521195.2; XM_005254498.3; XM_011521196.1; XM_017022369.1; XM_017022370.1	
CKMT2	Hs00176502_m1	NM_001825.2; NM_001099735.1; NM_001099736.1	68
DIO2	Hs00255341_m1	NM_013989.4; NM_000793.5; NM_001324462.1	88
PPIA	Hs99999904_m1	NM_021130.4; NM_001300981.1	98
CPT1B	Hs00189258_m1	NM_001145135.1; NM_001145134.1; NR_027928.2; NM_001145137.1; NM_004377.3; NM_152246.2; NM_152245.2	67
ZNF516	Hs00206187_m1	XM_017026097.1; XM_011526271.2; NM_014643.3; XM_011526272.2	84
NRIP1	Hs00940782_m1	XM_005261063.3	127
UCP1	Hs00222453_m1	XM_005263206.3; NM_021833.4	68
NR1H3	Hs00172885_m1	XM_005252709.1; XM_011519808.2; NM_001251935.1; XM_011519807.1; NM_001251934.1; XM_005252713.3; XM_006718113.1; XM_006718112.1; XM_006718115.1; XM_005252710.1; XM_005252716.3; XM_005252715.2; XM_017017058.1; NM_001130101.2; XM_017017057.1; XM_017017056.1; NM_001130102.2;	78

		XM_011519806.1; XM_011519805.2; XM_005252707.4; XM_006718116.1; NM_005693.3; XM_005252706.1; XM_005252705.1; XM_005252718.3	
SLC36A2	Hs00699197_m1	XM_006714756.3 ;NM_181776.2; XM_017009084.1	105
HPRT1	Hs02800695_m1	NM_000194.2	82
P2RX5	Hs00531938_m1	NM_002561.3; NM_001204519.1; NR_037928.1; NM_175080.2; NM_001204520.1	70
PPARG	Hs01115513_m1	XM_011533842.2; XM_011533844.1; NM_005037.5; NM_138711.3; NM_138712.3; NM_015869.4; XM_011533843.2; XM_011533841.2	90
PPARA	Hs00947536_m1	XM_011530240.2; XM_011530241.2; XM_011530242.2; XM_011530239.2; NM_001001928.2; NM_005036.4; XM_011530243.2; XM_011530244.2; XM_006724270.3; XM_006724269.3; XM_017028840.1; XM_005261655.3; XM_017028839.1; XM_011530245.2; XM_005261656.3	62
CEBPA	Hs00269972_s1	NM_001287424.1; NM_004364.4; NM_001287435.1; NM_001285829.1	77
PPARGC1 A	Hs00173304_m1	XM_011513769.2; XM_011513768.1; XM_011513767.2; XM_011513766.1; XM_011513765.2; XM_005248134.4; XM_005248132.1; XM_005248131.4; NM_013261.3; XM_011513771.1;	83

		XM_017007664.1; XM_011513770.2	
COL6A3	Hs00915125_m1	XM_011510574.1; NM_057166.4; NM_057164.4; XM_017003304.1; NM_004369.3; XM_005246066.1; XM_017003303.1; XM_006712253.1; NM_057167.3; XM_005246065.1; NM_057165.4	73
FOXC2	Hs00270951_s1	NM_005251.2	102
FABP4	Hs01086177_m1	NM_001442.2	96
CD36	Hs00354519_m1	NR_110501.1; NM_001289908.1; NM_001289909.1; NM_001289911.1; NM_001001547.2; NM_001001548.2; XM_005250714.1; XM_005250715.4; XM_005250713.1; NM_001127443.1; NM_001127444.1; NM_000072.3	83
SLC27A1	Hs01587911_m1	XM_011528003.2; XM_017026781.1; NM_198580.2; XM_011528000.1; XM_011528002.2; XM_011528001.2	120
FASN	Hs01005622_m1	XM_011523538.2; NM_004104.4	62
PLIN1	Hs00160173_m1	XM_005254934.4; NM_001145311.1; NM_002666.4	54
FFAR4	Hs00699184_m1	NM_181745.3; NM_001195755.1; XM_011539746.2	86
SLIT2	Hs01061407_m1	XM_006713986.3; NM_004787.3; XM_005248211.3; XM_017008845.1; NM_001289136.2; NM_001289135.2; XM_011513910.2; XM_011513909.2	66
PNPLA2	Hs00386101_m1	NM_020376.3; XM_006718265.3; XM_017018028.1; XM_006718266.3	116
LPL	Hs00173425_m1	NM_000237.2	103

PRKAA2	Hs00178903_m1	NM_006252.3;<br>XM_017001694.1;<br>XM_017001693.1;<br>XM_017001692.1	102
ELOVL6	Hs00907564_m1	NM_024090.2;<br>XM_011532235.2;<br>XM_011532234.2;<br>NM_001130721.1;<br>XM_011532233.2	75
ADIPOQ	Hs00605917_m1	NM_001177800.1;<br>NM_004797.3	71
IL6	Hs00174131_m1	XM_005249745.4;<br>NM_001318095.1;<br>NM_000600.4;<br>XM_011515390.2	95
LEP	Hs00174877_m1	NM_000230.2	74
FGF21	Hs00173927_m1	NM_019113.3	117
BMP7	Hs00233476_m1	NM_001719.2	73
BMP8B	Hs00236942_m1	NM_181809.3;<br>NM_001720.3;<br>XM_011542022.2	110
RETN	Hs00220767_m1	NM_020415.3;<br>NM_001193374.1	130
CFD	Hs00157263_m1	NM_001317335.1;<br>NM_001928.3	72
SREBF1	Hs00231674_m1	XM_005256772.4;<br>NM_004176.4	84
MLXIPL	Hs00975714_m1	NM_032953.2;<br>XM_011516278.1;<br>XM_011516277.1;<br>NR_134541.1;<br>NM_032952.2;<br>NM_032954.2;<br>XM_017012263.1;<br>XM_011516281.2;<br>XM_011516279.1;<br>NM_032951.2	56
INSR	Hs00961557_m1	XM_011527988.2;<br>NM_000208.3;<br>XM_011527989.2;<br>NM_001079817.2	66
IGF1R	Hs00609566_m1	XM_017022139.1;<br>XM_011521516.2;<br>XM_017022137.1;<br>XM_017022138.1;<br>XM_011521517.2;<br>NM_001291858.1;<br>NM_000875.4;<br>XM_017022136.1	64
RPLP0	Hs99999902_m1	NM_001002.3;<br>NM_053275.3	105
SLC2A4	Hs00168966_m1	NM_001042.2	89
BMPR1A	Hs01034913_g1	XM_011540104.2;	94

		XM_011540103.2; NM_004329.2	
NPR3	Hs01099013_m1	XM_011514049.2; NM_001204376.1; XM_011514048.2; NM_001204375.1; XM_011514047.1; XM_005248309.1; XM_017009492.1; NM_000908.3	65
NPR1	Hs01099745_m1	NM_000906.3; XM_017001374.1; XM_005245218.1	65
IL1B	Hs01555410_m1	XM_017003988.1; NM_000576.2	91
TNF	Hs00174128_m1	NM_000594.3	80
B2M	Hs99999907_m1	NM_004048.2	75

Supplementary Table 9 TaqMan OpenArray Real-Time PCR human primer details giving gene, ThermoFisher assay identification number, primer NCBI reference sequence and amplicon length.

Species	Gene	Commercial Vendor	Catalogue Number	Primer Reference Position
Human	*UCP1*	Qiagen (GeneGlobe)	PPH02223A-200	911 (NM_021833)
	PGC1α (PPARGC1a)		PPH00461F-200	2303 (NM_013261)
	CIDEA		PPH00899C-200	604 (NM_001279)
	CPT1b		PPH20905B-200	23 (NM_004377)
	ACADvl		PPH01732A-200	1530 (NM_000018)
	CYCS		PPH20675F-200	4466 (NM_018947)
	MCT1/SLC16A1		PPH09944F-200	1531 (NM_003051)
	PPARα		PPH01281B-200	668 (NM_005036)
	NDUFS1		PPH19871A-200	1768 (NM_005006)
Mouse	*Ucp1*		PPM05164B-200	1112 (NM_009463
	Pgc1α (Ppargc1α)		PPM03360I-200	2956 (NM_008904)
	Cidea		PPM03423C-200	455 (NM_007702)
	Cpt1b		PPM57688A-200	1910 (NM_009948)
	Acadvl		PPM04358E-200	1142 (NM_017366)
	Cycs		PPM28320A-200	295 (NM_007808)
	Mct1/Slc16a1		PPM25515A-200	1498 (NM_009196)
	Pparα		PPM03307C-200	1648 (NM_011144
	Ndufs1		PPM37821A-200	1866 (NM_145518)
	Bcat2		PPM26571A-200	977 (NM_009737)
	Bckdhb		PPM25713A-200	0 (NM_199195)
	Acads		PPM25953A-200	896 (NM_007383)
	Hadha		PPM32964A-200	2221 (NM_178878)
	Gss		PPM06172A-200	1323 (NM_008180)
	Ggct		PPM38268A-200	532 (NM_026637)

Supplementary Table 10 PCR primer details giving species, gene, commercial vendor, vendor catalogue number and primer reference position in NCBI reference sequence.

Supplementary References

1. Hoffer LJ, Taveroff A, Robitaille L, Mamer OA, Reimer ML. Alpha-keto and alpha-hydroxy branched-chain acid interrelationships in normal humans. *J Nutr* **123**, 1513-1521 (1993).

2. Hoffmann GF, Meier-Augenstein W, Stockler S, Surtees R, Rating D, Nyhan WL. Physiology and pathophysiology of organic acids in cerebrospinal fluid. *Journal of inherited metabolic disease* **16**, 648-669 (1993).

3. Avogaro A, Bier DM. Contribution of 3-hydroxyisobutyrate to the measurement of 3-hydroxybutyrate in human plasma: comparison of enzymatic and gas-liquid chromatography-mass spectrometry assays in normal and in diabetic subjects. *J Lipid Res* **30**, 1811-1817 (1989).

4. Engelke UF*, et al.* NMR spectroscopic studies on the late onset form of 3-methylglutaconic aciduria type I and other defects in leucine metabolism. *NMR in biomedicine* **19**, 271-278 (2006).

5. Friesen RW, Novak EM, Hasman D, Innis SM. Relationship of dimethylglycine, choline, and betaine with oxoproline in plasma of pregnant women and their newborn infants. *J Nutr* **137**, 2641-2646 (2007).
