# Supplementary material for: Brown and beige adipose tissue regulate systemic metabolism through a metabolite interorgan signaling axis
Source: Nat Commun. 2021 Mar 26;12:1905. doi: 10.1038/s41467-021-22272-3 (PMC7998027; doi:10.1038/s41467-021-22272-3)
Supplement: Supplementary file 3 — Reporting Summary [file 41467_2021_22272_MOESM3_ESM.pdf]

nature research

Corresponding author(s): Dr Lee D Roberts

Last updated by author(s): Feb 8, 2021

Reporting Summary

Nature Research wishes to improve the reproducibility of the work that we publish. This form provides structure for consistency and transparency in reporting. For further information on Nature Research policies, see our Editorial Policies and the Editorial Policy Checklist.

Statistics

For all statistical analyses, confirm that the following items are present in the figure legend, table legend, main text, or Methods section.

n/a	Confirmed	
☐	☒	The exact sample size (*n*) for each experimental group/condition, given as a discrete number and unit of measurement
☐	☒	A statement on whether measurements were taken from distinct samples or whether the same sample was measured repeatedly
☐	☒	The statistical test(s) used AND whether they are one- or two-sided<br>*Only common tests should be described solely by name; describe more complex techniques in the Methods section.*
☐	☒	A description of all covariates tested
☐	☒	A description of any assumptions or corrections, such as tests of normality and adjustment for multiple comparisons
☐	☒	A full description of the statistical parameters including central tendency (e.g. means) or other basic estimates (e.g. regression coefficient) AND variation (e.g. standard deviation) or associated estimates of uncertainty (e.g. confidence intervals)
☐	☒	For null hypothesis testing, the test statistic (e.g. *F*, *t*, *r*) with confidence intervals, effect sizes, degrees of freedom and *P* value noted<br>*Give P values as exact values whenever suitable.*
☒	☐	For Bayesian analysis, information on the choice of priors and Markov chain Monte Carlo settings
☒	☐	For hierarchical and complex designs, identification of the appropriate level for tests and full reporting of outcomes
☒	☐	Estimates of effect sizes (e.g. Cohen's *d*, Pearson's *r*), indicating how they were calculated

Our web collection on statistics for biologists contains articles on many of the points above.

Software and code

Policy information about availability of computer code

Data collection	Mass spectrometry metabolomic data was collected in Analyst (ABSciex, version 1.6.2) or Xcalibur (Thermo Scientific, version 2.2). 13C substrate labelling data was collected in Xcalibur (Thermo Scientific, version 2.2). PET/CT data was collected using Albira Reconstructor software in PMOD (version 3.807, PMOD Technologies, Zurich, Switzerland). RT-qPCR data was collected using StepOne™ Software (version 2.1 Applied Biosystems). Gene expression array data was collected using Biosystems™ QuantStudio™ software version 1.2.10 (ThermoFisher). Confocal images were collected and analysed using Columbus software version 2.9.1 (PerkinElmer). Indirect calorimetry data was collected using Oxymax (version 5.37.05, Columbus Instruments). Respirometry data was collected using Si 782 System (version 4.1 Strathkelvin Instruments) and Oxygraph 2k DatLab software (version 6.1, OROBOROS Instruments). Histology images were collected using Zen 2 Pro (version 2.0, ZEISS).
Data analysis	Multivariate data analysis was performed using SIMCA-P+ 13.0 (Umetrics AB, Umeå, Sweden) and Metaboanalyst version 4.0 (https://www.metaboanalyst.ca/MetaboAnalyst/Secure/analysis/AnalysisView.xhtml). Indirect calorimetry data was analyzed and p-values were calculated using ANCOVA / Generalized Linear Model with body mass as a covariate using CalR (version 1.1) (https://calrapp.org/). Univariate data analysis was performed using Prism Graphpad (version 6.02). Confocal images were analysed using Columbus software version 2.9.1 (PerkinElmer). Data analysis of RNA-Seq data was performed using R package edgeR v3.8.6. Gene expression array data was analyzed in the Thermo Fisher Cloud Relative Quantification App (3.4.1-PCR-build3 2017-09-26). Mitochondrial respiration profiles were assessed using Seahorse Wave Software (version 2.6.1) (Agilent Technologies, Waldbronn, Germany).

For manuscripts utilizing custom algorithms or software that are central to the research but not yet described in published literature, software must be made available to editors and reviewers. We strongly encourage code deposition in a community repository (e.g. GitHub). See the Nature Research guidelines for submitting code & software for further information.

Data

Policy information about availability of data

All manuscripts must include a data availability statement. This statement should provide the following information, where applicable:

- Accession codes, unique identifiers, or web links for publicly available datasets
- A list of figures that have associated raw data
- A description of any restrictions on data availability

RNA-Seq data associated with this study is available from Gene Expression Omnibus [GSE129153] (https://www.ncbi.nlm.nih.gov/geo/query/acc.cgi?acc=GSE129153). Human GWAS data is available from The Type 2 Diabetes Knowledge Portal (http://www.type2diabetesgenetics.org/). Mass spectrometry metabolomics data associated with this study is available from the EBI MetaboLights Database [MTBLS2436]. A Source Data are provided as a Source Data file containing data on all figures within this manuscript.

Field-specific reporting

Please select the one below that is the best fit for your research. If you are not sure, read the appropriate sections before making your selection.

- [x] Life sciences
- [ ] Behavioural & social sciences
- [ ] Ecological, evolutionary & environmental sciences

For a reference copy of the document with all sections, see nature.com/documents/nr-reporting-summary-flat.pdf

Life sciences study design

All studies must disclose on these points even when the disclosure is negative.

Sample size	Sample sizes for animal experiments (primary outcomes: change in weight and energy expenditure) were calculated using power calculations. Standard deviations were based on previous studies or on the available literature. Power calculations were made using beta = 0.8 and α = 0.05. Where sample sizes were not calculated sample size was considered adequate based on the size and reproducibility of between group differences.
Data exclusions	Data was only excluded where experiments failed (based on positive or negative controls, animals or cells failed to meet experiment endpoint or variance of internal standards), or where limited material (human tissues) were exhausted.
Replication	Replicate experiments were successful. Experiments were replicated a minimum of twice with the exception of human volunteer studies and sufficiently powered animal studies.
Randomization	All samples were randomly assigned to experimental groups. Animals were weight matched and randomly assigned to experimental groups. Cells in culture wells were randomly assigned to study groups.
Blinding	For animal studies researchers were blinded to experimental groups by core technicians in the animal facility to avoid bias and aid reproducibility - e.g. PET/CT studies. Experimentalists were blinded to study groups for cell culture endpoint data collection and analysis. Experimentalists were blinded to human patient data including BMI for human tissue analyses by clinical and research staff.

Reporting for specific materials, systems and methods

We require information from authors about some types of materials, experimental systems and methods used in many studies. Here, indicate whether each material, system or method listed is relevant to your study. If you are not sure if a list item applies to your research, read the appropriate section before selecting a response.

Materials & experimental systems

n/a	Involved in the study	
[ ]	[x]	Antibodies
[ ]	[x]	Eukaryotic cell lines
[x]	[ ]	Palaeontology and archaeology
[ ]	[x]	Animals and other organisms
[ ]	[x]	Human research participants
[x]	[ ]	Clinical data
[x]	[ ]	Dual use research of concern

Methods

n/a	Involved in the study	
[x]	[ ]	ChIP-seq
[x]	[ ]	Flow cytometry
[x]	[ ]	MRI-based neuroimaging

Antibodies

Antibodies used	Mouse anti-human/anti-mouse UCP-1 monoclonal antibody (R&D Systems MAB6158, Minneapolis, USA)<br>Anti-UCP1 primary antibody (abcam ab23841, UK)<br>Alexa455 AffiniPure donkey anti-mouse IgG (H+L) antibody (Jackson ImmunoResearch 715-545-150, West Grove, USA)

Validation	Anti-UCP1 primary antibody (abcam ab23841, UK): This antibody is validated for Western blotting and Immunohistochemistry (https://www.abcam.com/ucp1-antibody-ab23841.html)<br>Mouse anti-human/anti-mouse UCP-1 monoclonal antibody (R&D Systems MAB6158, Minneapolis, USA): This antibody is validated for Western blotting and Immunohistochemistry amongst other applications https://www.rndsystems.com/products/human-mouse-ucp1-antibody-536435_mab6158.<br>Alexa455 AffiniPure donkey anti-mouse IgG (H+L) antibody is validated for immunofluorescence procedures (https://www.jacksonimmuno.com/catalog/products/715-545-150).

Eukaryotic cell lines

Policy information about cell lines

Cell line source(s)	C2C12 cells were purchased from the European Collection of Authenticated Cell Culture Operated by Public Health England.<br>Adult human skeletal myoblasts were purchased from Cell applications Inc. (Cat no. 150-05a).<br>Human white primary preadipocytes were purchased from PromoCell, Heidelberg, Germany (Cat no. C-12735).<br>Immortalized Human white adipocytes were obtained from Dr Yu-Hua Tseng by AstraZeneca, Aaron Klug Building, Granta Park, Cambridge, CB21 6GH, UK in 2016
Authentication	Human primary adipocytes are authenticated by the suppliers. In addition, both human primary (PromoCell, Heidelberg, Germany, Cat no. C-12735) and immortalized adipocytes are tested for cell morphology, adherence rate, and cell viability. Growth performance is tested through multiple passages under culture conditions. Preadipocytes are tested for their capacity to differentiate into mature adipocytes using morphological and gene/protein expression markers.<br><br>Human primary skeletal myoblasts (Cell applications Inc. Cat no. 150-05a) are authenticated by the suppliers. In addition myoblasts are tested for their ability to attach, spread and proliferate in culture conditions. The cells are authenticated for their capacity to form multinucleated myotubes using morphological and gene/protein expression markers following differentiation.<br><br>The C2C12 cell line was purchased from Sigma Aldrich (Cat no 91031101) and authenticated by the providers. In addition we authenticate that C2C12 cells on differentiation express muscle specific markers and exhibit a myotube morphology and have previously published details that these cells express these markers: Ashmore et al. Nitrate enhances skeletal muscle fatty acid oxidation via a nitric oxide-cGMP-PPAR-mediated mechanism. BMC Biol. 13:110.
Mycoplasma contamination	All cells have been tested for mycoplasma contamination by the providers (negative results).
Commonly misidentified lines<br>(See ICLAC register)	No commonly misidentified cell lines were used in this study.

Animals and other organisms

Policy information about studies involving animals; ARRIVE guidelines recommended for reporting animal research

Laboratory animals	For primary adipocyte isolation 6–10-week-old C57BL/6J male mice were used (Charles River). For metabolite treatment studies six-week-old male C57BL6/J mice were used (Charles River). These mice were were housed in conventional cages at room temperature with humidity maintained at 40 – 60% and a 12 hour light/dark photoperiod.<br><br>For temperature conditioning studies 12 week old male C57BL6/J mice were used. Animals were housed in a specific pathogen free facility with 12 hour light and 12 hour dark cycles and humidity maintained at 40 – 60%. Mice underwent thermal adaptation at 12 weeks of age. One group was placed at 8˚C for 4 weeks, a second group was maintained at room temperature for 3 weeks then placed at 8˚C for one week, a further group was placed at 28˚C for 4 weeks and a final group was maintained at room temperature for 4 weeks (21-23˚C). The mice were terminated at 16 weeks of age.
Wild animals	The study did not involve wild animals.
Field-collected samples	The study did not involve animals collected from the field.
Ethics oversight	All animal studies were regulated under the Animals (Scientific Procedures) Act 1986. For the cold conditioned studies these were also under Amendment Regulations 2012 following ethical review by the University of Cambridge Animal Welfare and Ethical Review Body (AWERB). All procedures were carried out in accordance with U.K. Home Office protocols by a personal license holder under a Home Office Project Licence.

Note that full information on the approval of the study protocol must also be provided in the manuscript.

Human research participants

Policy information about studies involving human research participants

Population characteristics	Forty two patients were recruited for this study. The primary outcome was mass spectrometry analysis of metabolites in subcutaneous white adipose tissue biopsies and correlation to body mass index. Where sufficient tissue remained, a secondary assessment was analysis of gene expression in subcutaneous white adipose tissue (30 patients). Inclusion criteria for subjects included:<br>Normal cardiac function on echocardiography

	No symptoms or past diagnosis of heart failure<br>Able and willing to give informed consent<br><br>Exclusion criteria for subjects included:<br>Unable or unwilling to give consent<br>Symptoms possibly due to heart failure<br>Current or previous diagnosis of cancer, inflammatory or musculoskeletal disease (e.g. rheumatoid arthritis)<br>Ongoing infection or sepsis, type 1 or type 2 diabetes mellitus, heart failure.<br>The recruited patient demographics are: age = 74.9 +/- 1.3 (years), Sex = 78.6% Male (33/42), weight = 84.5 +/- 1.3 (Kg), Body Mass Index = 27.6 +/- 0.7. Data is Mean +/- SEM.
Recruitment	Eligible and consecutive patients undergoing routine de-novo pacemaker implantation at Leeds General Infirmary, Leeds Teaching Hospital Trust, UK volunteered to participate in the study and provided written consent. Self-selection bias is unlikely to be an important factor. All patients fulfilling the inclusion criteria are approached consecutively during the clinical list. Given the nature of the sampling process, which is pain free, and therefore goes unnoticed during the routine clinical procedure, patients rarely decline to participate. In the time frame of the recruitment to this study we had one patient decline to participate.
Ethics oversight	The study is approved by the Leeds West Research Ethics Committee (11/YH/0291) and Leeds Teaching Hospitals Trust R&D committee (CD11/10015) and conforms to the Declaration of Helsinki.

Note that full information on the approval of the study protocol must also be provided in the manuscript.
